# Characterization of the Transcriptome and Gene Expression of Tetraploid Black Locust Cuttings in Response to Etiolation

**DOI:** 10.3390/genes8120345

**Published:** 2017-11-24

**Authors:** Nan Lu, Li Dai, Zijing Luo, Shaoming Wang, Yanzhong Wen, Hongjing Duan, Rongxuan Hou, Yuhan Sun, Yun Li

**Affiliations:** 1 Beijing Advanced Innovation Center for Tree Breeding by Molecular Design, National Engineering Laboratory for Tree Breeding, Key Laboratory of Genetics and Breeding in Forest Trees and Ornamental Plants of Ministry of Education, College of Biological Sciences and Technology, Beijing Forestry University, Beijing 100083, China; ln_890110@163.com (N.L.); daili.jiayou@163.com (L.D.); luozijingbjfu@163.com (Z.L.); duan673356712@126.com (H.D.); hrxvshzy@163.com (R.H.); syh831008@163.com (Y.S.); 2 State Key Laboratory of Forest Genetics and Tree Breeding, Key Laboratory of Tree Breeding and Cultivation of State Forestry Administration, Research Institute of Forestry, Chinese Academy of Forestry, Beijing 100091, China; 3 The State-Owned Luoning County Lv Cun Forest Farm, Luoning 471711, China; lclc873@163.com; 4 The State-Owned Ji County Nursery of Forests, Linfen 042200, China; jxzzywyz@163.com

**Keywords:** Tetraploid *Robinia pseudoacacia* L., etiolation, transcriptome, phytohormone, peroxidase activity

## Abstract

Etiolation (a process of growing plants in partial or complete absence of light) promotes adventitious root formation in tetraploid black locust (*Robinia pseudoacacia* L.) cuttings. We investigated the mechanism underlying how etiolation treatment promotes adventitious root formation in tetraploid black locust and assessed global transcriptional changes after etiolation treatment. Solexa paired-end sequencing of complementary DNAs (cDNAs) from control (non-etiolated, NE) and etiolated (E) samples resulted in 107,564 unigenes. In total, 52,590 transcripts were annotated and 474 transcripts (211 upregulated and 263 downregulated) potentially involved in etiolation were differentially regulated. These genes were associated with hormone metabolism and response, photosynthesis, signaling pathways, and starch and sucrose metabolism. In addition, we also found significant differences of phytohormone contents, activity of following enzymes i.e., peroxidase, polyphenol oxidase and indole acetic acid oxidase between NE and E tissues during some cottage periods. The genes responsive to etiolation stimulus identified in this study will provide the base for further understanding how etiolation triggers adventitious roots formation in tetraploid black locus.

## 1. Introduction

Tetraploid black locust (*Robinia pseudoacacia* L.) is produced by artificially doubling the chromosomes of a diploid black locust, and the species was first imported from South Korea into China in 1997 [1]. Compared to the normal diploid *Robinia pseudoacacia*, tetraploid *R. pseudoacacia* has a significantly higher yield with larger leaves and higher leaf protein content. It is polyanthus, long blossoming, and suitable for feeding and beekeeping. Additionally, tetraploid *R. pseudoacacia* is fast growing and able to tolerate harsh environments including salt, drought, and nutrient deficiencies. Thus, it has high ecological value and is widely planted to improve the soil [2].

Stem cuttings are the main method used to propagate tetraploid black locust because it is the most economical and simplest vegetative propagation method. However, the low rooting rate of traditional tetraploid *R. pseudoacacia* is a problem [3]. Although tetraploid black locust is a hard-rooting species, we previously improved the rooting rate of tetraploid *R. pseudoacacia* stems using etiolated (E) juvenile branches as cutting material, causing new buds or juvenile branches to develop without chlorophyll by growing without exposure to sunlight before cutting [4]. This is a common way to improve the rooting rate of cuttings and has beneficial effects on the rooting of avocados, apples, maples, and other plants [5,6,7].

The mechanism by which etiolation promotes rooting in tetraploid black locust juvenile cutting stems remains unclear. Some studies have indicated that adventitious root formation and elongation of E seedlings are induced by ethylene [7] or due to increased mobilization of carbohydrates from starch [8]. There is also evidence that etiolation improves the rooting rate of cuttings by affecting the levels of sugars and some rooting co-factors [9]. Deficiencies in substances such as strigolactones and cytokinin, which restrain adventitious root development, may be another reason [10]. In a previous study, we analyzed the differential expression of proteins between E and control (non-etiolated, NE) samples during different rooting periods and found 58 differentially expressed proteins using two-dimensional electrophoresis gel staining and image analysis [11]. However, the key molecular factors that promote rooting of E branches remain unclear. We compared the transcriptomes of NE juvenile branches using Solexa paired-end RNA-sequencing technology and de novo transcriptome assembly to further study the genes associated with the etiolation response, and to screen genes that might participate in rooting of the tetraploid black locust. Annotation of the completely expressed gene catalogue is reported based on comparative analyses using published datasets. The altered expression levels of these transcripts implicated in the etiolation response in tetraploid black locust juvenile branches were analyzed, and the potential roles of these transcripts are discussed.

To determine the physiology and biochemical changes in E cuttings, we also detected the levels of indole acetic acid oxidase (IAAO), polyphenol oxidase enzyme (PPO), and peroxidase (POD), as well as four endogenous hormones including indole acetic acid (IAA), abscisic acid (ABA), gibberellic acid (GA3), and zeatin riboside (ZR) in tetraploid *R. pseudoacacia* E and NE juvenile branches after cutting [10], which are associated with adventitious root formation. The goal of this study was to provide a theoretical basis for the molecular mechanisms underlying etiolated promotion of adventitious root development of softwood cuttings in tetraploid black locust.

## 2. Materials and Methods

### 2.1. Plant Material and Etiolation Procedures

**Figure 1:**
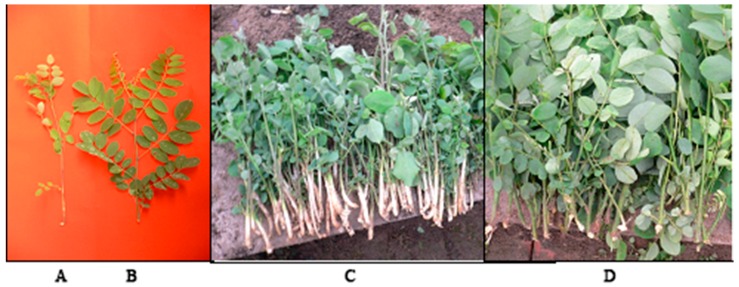
The photos of etiolated and non-etiolated tetraploid *Robinia pseudoacacia* cuttings. (**A**,**C**) Etiolated tetraploid *R. pseudoacacia*; (**B**,**D**) Non-etiolated tetraploid *R. pseudoacacia* cuttings.

Clonally propagated stems of tetraploid *R. pseudoacacia* K4 were collected from Lv Cun National Forest Farm (Luoning, Henan, China) in April 2014. The stems were cut into 30–40 cm lengths with diameters of ≈3 cm. The plants were grown in quadrate plastic pots (length = 80 cm, width = 60 cm, height = 20 cm) with a mixture of vermiculite:perlite:sand (1:1:1 *v*/*v*) at depths of 0.5 and 6 cm from the surface of the substrate in a greenhouse at Beijing Forestry University (12/12 h diurnal light/dark cycles, temperature of 25 °C during the light cycle and 18 °C during the dark cycle, 6.7 lumen output flux, 70% relative humidity). Juvenile branches that sprouted from stems buried at a depth of 0.5 cm were NE, whereas those 6 cm deep were E. The plants were watered every two days. After approximately 20 days, when the juvenile branches were ≈15 cm high, E and NE juvenile branches (Figure 1) were sheared from the stems and cuttings in the greenhouse at 1 h, 1 day (d), 3 d, 5 d, 7 d, 9 d, and 11 d (treated with 2 g/L indole-3-butyric acid (IBA) solution for 30 s before cutting). At each time point, 2 cm material from the base of the cuttings was stripped, snap-frozen in liquid nitrogen, and stored at −80 °C (each treatment was randomly sampled three times).

### 2.2. Plant Hormone Content Measurements

Total IAA, ABA, GA3, and ZR content in NE and E samples at each time point were extracted and purified using the following method. Fresh tissues (0.5–1.0 g) were ground to a powder with a mortar and pestle in liquid nitrogen. The samples were extracted in 2 mL methanol containing 0.01% (*w*/*v*) butylated hydroxytoluene overnight at 4 °C. Then, the sample was centrifuged (1370× *g*, 8 min), and the supernatant was collected and dried under a stream of air. Samples were purified using a C-18 Sep Pack column (Waters, Milford, MA, USA) that was flushed with 1 mL 100% (*v*/*v*) methanol followed by 1 mL 10% (*v*/*v*) methanol. The samples were dissolved in 10% methanol and loaded onto the column. The column was washed with 1 mL 10% (*v*/*v*) methanol twice and flushed. Samples were eluted with 1 mL 80% (*v*/*v*) methanol and dried under a stream of air. The samples were dissolved in methanol (sample from 1 g fresh tissue was dissolved in 2 mL methanol) and diluted with Tris-buffered saline. Indirect enzyme-linked immunosorbent assays (ELISA) were used to assay phytohormones according to Norman et al. [12] for ABA, and Yang et al. [13] for IAA, GA, and ZR. The concentrations of each phytohormone were calculated based on a standard curve and expressed as ng g^−1^ fresh weight.

### 2.3. Analysis of Phenylene Oxide, Peroxidase and Indole Acetic Acid Oxidase

The enzyme activities of the samples were measured with the method described by Zhang [14]. The samples (0.2 g) were ground to a homogenate in 1 mL cold phosphate buffer (20 mM, pH 6.0) and a small amount of quartz sand in a mortar in an ice bath. After the homogenate was diluted to 2 mL, the supernatant was collected by centrifugation (1790× *g*, 20 min, 4 °C) and readjusted to constant volume. To analyze IAAO, 1 mL enzyme solution was added to the reaction solution (1 mL 1 × 10^*−*3^ M MnCl_2_, 1 mL 1 × 10^−3^ M 2,4-dichlorophenol, 2 mL 1 × 10^−3^ M IAA and 5 mL phosphate buffer) and mixed in tube 1. The same volume of phosphate buffer was added to the same reaction system in tube 2. Distilled water was added to tube 3 as a control. Then, 1 mL of each mixed solution was added to 2 mL FeCl_3_ perchloric acid reagent, shaken, and incubated at 30 °C for 30 min in the dark. The absorbance value was determined at 530 nm. To analyze PPO, after 3 mL phosphate buffer (pH 6.0) and 0.1 mL enzyme solution were reacted for 1 min, 1 mL pyrocatechol (0.08 M) was added and shaken. The absorbance value was determined at 535 nm. To analyze POD, after 0.1 mL enzyme solution was added to the reaction solution (2.9 mL 0.05 M phosphate buffer, 1.0 mL 2% H_2_O_2_, 1.0 mL 0.05 M guaiacol) in a tube, it was incubated in a water bath at 30 °C for 3 min. Then, 1.0 mL 2% H_2_O_2_ was added and incubated for a further 2 min. The absorbance value was determined at 470 nm.

### 2.4. RNA Extraction and Deep Sequencing

Three seedlings were randomly selected from NE and E tissues (1 h). Total RNA was extracted from the tissues using the RNA Easyspin Isolation System (Aidlab Biotech, Beijing, China), according to the manufacturer’s protocol. RNA quality was confirmed with a 2100 Bioanalyzer (Agilent Technologies, Santa Clara, CA, USA) and RNA integrity number >8.0. To prepare complementary DNA (cDNA), we used a pooled RNA mixture containing 60 μg RNA from each sample. Each sample had three biological replicates. Illumina sequencing was conducted using the Solexa mRNA-Seq platform (Illumina, San Diego, CA, USA). The RNA was treated with oligo(dT) to isolate poly(A) mRNA after isolating total RNA from the samples. Sequencing libraries were prepared using the TruSeq RNA sample preparation kit (ver. 2; Illumina). The reverse transcription reaction was depurinated with a QiaQuick PCR extraction kit (Qiagen, Hilden, Germany) and resolved with an elution buffer for end repair by adding poly(A). The library was sequenced paired end 100 nucleotides (nt) mulitplex using the Illumina HiSeq 2000. Raw reads produced from sequencing machines contain low-quality reads that negatively affect subsequent bioinformatics analyses, so we discarded the reads with adapters, those with more than 5% unknown nucleotides, and those of low quality (≤20% of the bases with a quality score ≤10) using an in-house Perl script. The average proportion of clean reads in each sample was 47.37–95.1%. The clean reads were used for further analyses. RNA-sequencing (RNA-Seq) data can be downloaded from NCBI BioProjects PRJNA354493.

### 2.5. Analysis of Illumina Transcriptome Sequencing Results

De novo assembly was conducted using scaffolding contig methods with CLC Genomics Workbench (ver. 6.0.4), a word size of 45 with default parameters, and a minimum contig length ≥300. The assembled de novo sequences were designated as primary unigenes. The primary unigenes from UniGene were assembled using CAP3 expressed sequence tag (EST), yielding the final unigenes. The assembled final unigenes were used for BLASTx searches (*E*-value < 1 × 10^−5^) against the Swiss-Prot protein database [15], which is the largest annotated protein database. We used the Blast2GO program to functionally annotate sequences and assign Gene Ontology (GO) terms [16]. To predict and classify possible functions, unigene sequences were aligned to 25 Clusters of Orthologous Groups (COGs) in the COG database [17]. Kyoto Encyclopaedia of Genes and Genomes Pathway [18] annotations were performed according to the KEGG database using BLASTx (*E*-value threshold = 10^−5^).

### 2.6. Differential Expression Analysis and Functional Enrichment

The clean read expression distribution was used to evaluate normality of the data. All of the clean reads were mapped to our transcriptome reference sequences using SOAPaligner/soap2, and mismatches as small as 1 base pair (bp) were considered. The number of unambiguous clean reads for each gene was calculated and then normalized to the number of reads per kilobase per million clean reads (RPKM), uniquely aligning within each sample [19]. A rigorous algorithm to identify differentially expressed genes (DEGs) was developed, based on the method of Audic and Claverie [20]. The false discovery rate (FDR) was used to determine the threshold *p*-value in multiple tests and analyses. Two parameters (RPKM and FDR) were used to identify DEGs between the two samples: a fold-change not less than 4 (an absolute value of log2 ratio (E/NE) ≥ 2) and a FDR adjustment with a significance level of 0.05 [21]. GO and pathway enrichment were performed with a cut-off *p*-value of 0.05 compared to the whole transcriptome. For further analyses, we used only DEGs with a minimum four-fold change in expression.

### 2.7. Statistical Analyses

Means of three replications were calculated. Data are presented as mean ± standard deviation. The data were analyzed with one-way analysis of variance using SPSS software (ver. 19.0; SPSS Inc., Chicago, IL, USA).

## 3. Results

### 3.1. Indoleacetic Acid, Abscisic Acid, Gibberellin, and Zeatin Riboside Content

**Figure 2:**
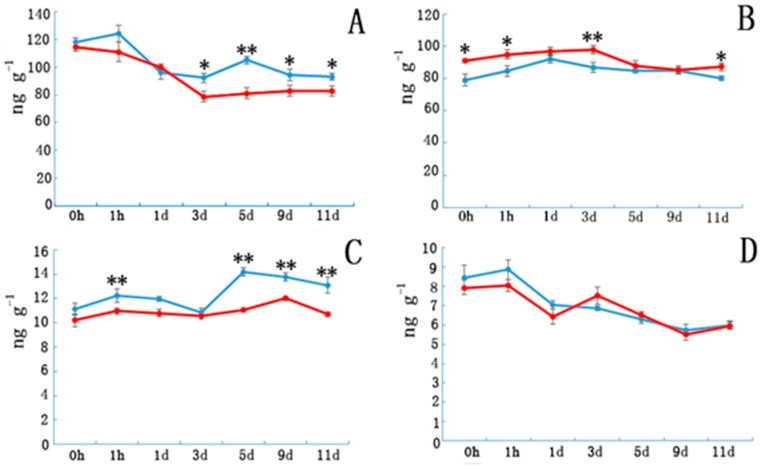
The plant hormone content in etiolated and non-etiolated samples during the different cutting periods. (**A**) Indole acetic acid (IAA), (**B**) abscisic acid (ABA), (**C**) gibberellic acid (GA_3_), and (**D**) zeatin riboside (ZR) contents (ng/g) at different time points. Etiolated (E) treatment (blue) and control (non-etiolated, NE) treatment (red). * and ** denotes a significant difference (0.01 < *p* < 0.05, α = 0.05) or an extremely significant difference (*p* < 0.01, α = 0.05) between treatments. Error bars represent the standard deviation.

Etiolated samples and control IAA levels (Figure 2A) sharply decreased in the first three days and subsequently became stable in NE tissue. However, IAA content in E tissue increased from days 3 to 5 and then decreased from days 5 to 9. Indoleacetic acid (IAA) contents in E tissues were significantly higher than in controls after day 5. Abscisic acid(ABA) contents (Figure 2B) in the E samples increased during the first few days and then decreased, whereas ABA content in NE samples increased slowly during the first three days, and then began to decrease. Gibberellin*3* (GA_3_) contents in E samples were significantly higher than those in the controls during the first hour after cutting, and then decreased until day 3 before peaking on day 5. Except for day 3, the GA_3_ contents in E samples were significantly higher than those in the controls. Etiolation did not significantly affect the ZR content.

### 3.2. Peroxidase, Phenylene Oxide and Indole Acetic Acid Oxidase Activity

**Figure 3:**
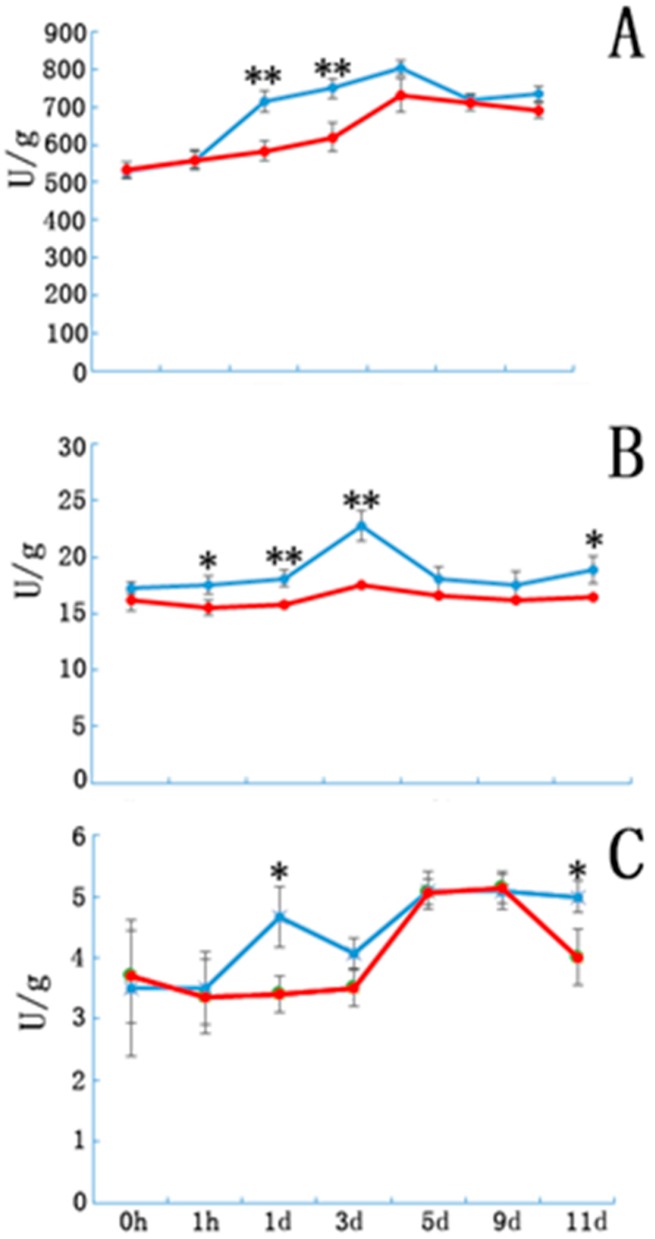
The peroxidase, indole acetic acid oxidase and polyphenol oxidase enzyme activity in etiolated and non-etiolated samples during the different cutting periods. (**A**) Peroxidase, (**B**) indole acetic acid oxidase, and (**C**) polyphenol oxidase enzyme activities (U/g). Etiolated (E) treatment (blue) and control (non-etiolated, NE) treatment (red). * and ** denote significant differences (0.01 < *p* < 0.05, α = 0.05) or extremely significant differences (*p* < 0.01, α = 0.05) between treatments. Error bars represent the standard deviation.

Peroxidase, IAAO, and PPO activity in the E and NE samples are shown in Figure 3. Peroxidase activity in both groups showed a sustained increase until day 5 of cutting. Peroxidase activity in the E samples was significantly higher than that in the controls on days 1 and 3. Although the variation in the trends of IAAO and PPO activities were similar in the E and NE samples, enzymatic activity in the E samples was significantly higher at certain time points after cutting (Figure 3B,C).

### 3.3. Deep Sequencing and de Novo Assembly

To screen the specifically expressed unigenes in E cuttings, a cDNA sample was prepared from equal RNA isolated from the base of the cuttings for the libraries (three replicates), corresponding to the NE tissues, which were sequenced. An average of 62,315,544 and 61,420,516 raw reads were generated from the NE and E tissue samples by RNA deep sequencing, respectively (Supplementary Table S1). We discarded the low-quality reads, which contained adapters and unknown or low-quality bases. After cleaning the data, we obtained 113,233 contigs with a length >300 bp and 107,564 primary unigenes. After local assembly with the unmapped ends to fill in the small gaps within the scaffolds, de novo assembly yielded 107,564 unigenes with an average length of 755 bp (Supplementary Table S2).

### 3.4. Sequence Alignment and Functional Annotation of the Transcriptome

**Figure 4:**
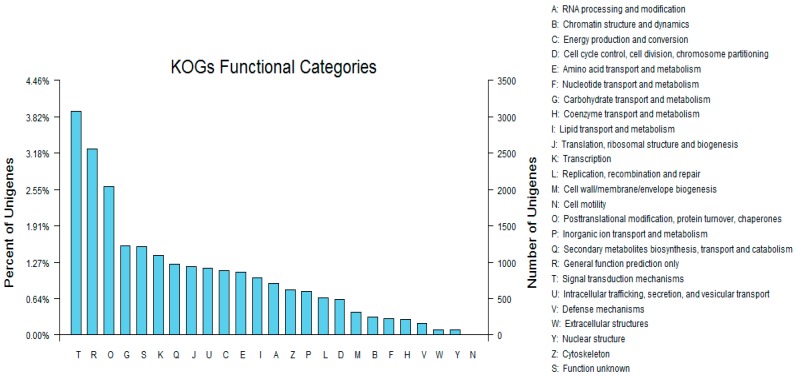
Clusters of orthologous groups (COG) classifications in tetraploid *R. pseudoacacia*.

**Figure 5:**
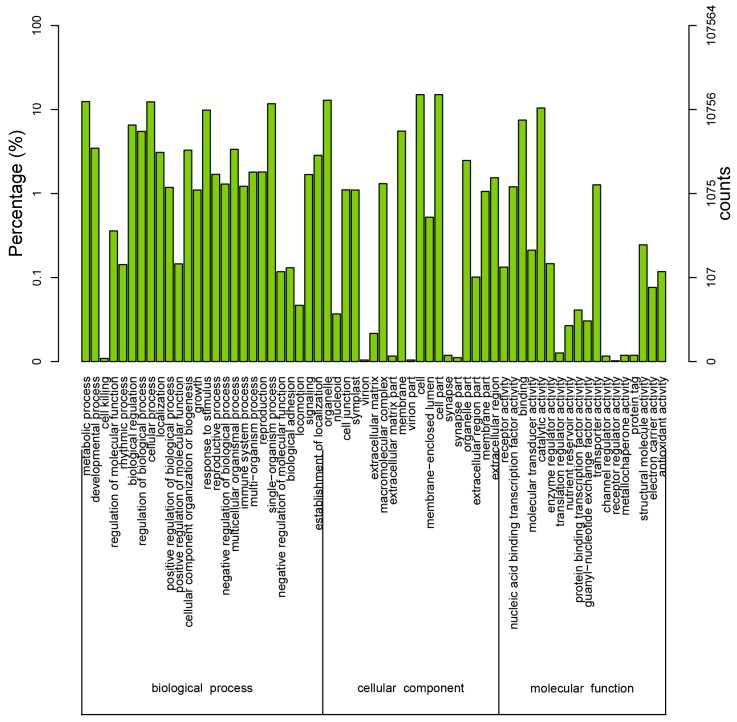
Histogram of Gene Ontology (GO) classifications. The results are classified into three main categories: biological process, cellular component, and molecular function. The *y*-axis on the left side indicates the percent of genes in a category, and the *y*-axis on the right side represents the number of genes.

To further evaluate the effectiveness of our annotation process, we randomly searched the annotated sequences for genes with COG classifications. Of the sequences, 29,005 had a COG classification. Among the 25 COG categories, the cluster for “signal transduction mechanisms” (3079, 10.62%) represented the largest group, followed by “general function prediction only” (2559, 8.82%) and “post-translational modification, protein turnover, chaperones” (2045, 7.05%). The “nuclear structure” (72, 0.25%) and “cell motility” (10, 0.03%) categories were the smallest groups (Figure 4). The results indicated that 52,590 (48.89%) genes had at least one hit in the databases: 36.47% of the hits were from *Glycine max*. In total, 21,756 (41.36%) of the genes were categorized into 55 GO terms (level 2), with mapping to one or more GO terms. Of the GO terms, “metabolic process” (14.51%) and “cellular process” (14.25%) were dominant within the biological process terms, while “cell” (27.66%) and “cell part” (27.66%) were dominant in the cellular component terms. In addition, “catalytic activity” (44.94%) and “binding” (34.23%) were dominant in the molecular function terms (Figure 5). In our study, 2383 unigenes were assigned to 287 pathways. The top 28 pathways containing the highest number of genes, along with the number of unigenes associated with each pathway, are shown in Supplementary Table S3. The highest number of unigenes was associated with the category “metabolic pathways” (681). The other four major pathways were “biosynthesis of secondary metabolites” (376), “carbon metabolism” (106), “biosynthesis of amino acids” (102), and “protein processing in the endoplasmic reticulum” (114).

**Figure 6:**
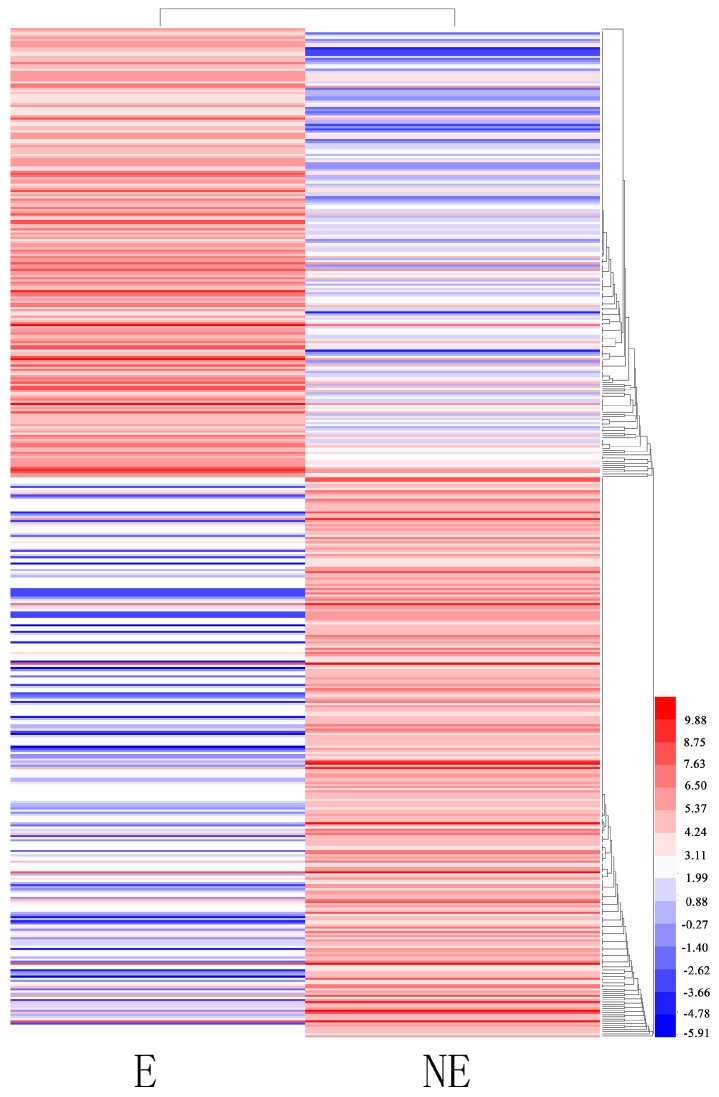
Clustering analysis of the total differentially expressed genes (DEGs) in etiolated (E) and control (non-etioleted, NE) plants based on their expression profiles obtained from RNA-sequencing (RNA-Seq) experiments. Color scale corresponds to the log2 (reads per kilobase of transcript per million mapped reads, RPKM) of different genes in the samples.

Differentially expressed transcript (DET) analyses were conducted for the NE and E samples to identify genes in the juvenile branches of tetraploid black locust that were specifically induced or suppressed in response to etiolation. In total, 474 transcripts were identified as being differentially expressed (FDR ≤ 0.05 and an absolute value of log2 ratio (E/NE) ≥ 2): 211 transcripts were upregulated, and 262 were downregulated. In total, 386 DETs were expressed in both libraries: 86 DETs were expressed only in the NE library (Figure 6). Enrichment analyses were conducted to classify the biological function of the DETs. The results indicated that 365 DETs could be annotated in GO terms. Among the GO categories, “chloroplast” (102), “cytoplasm” (59) and “molecular function” (49) were significantly enriched (Supplementary Table S4). The GO analysis of the downregulated genes showed that they were related mainly to the cytosol, nucleus, molecular function, plasma membrane and the extracellular region (*p* < 0.05). The upregulated genes were related primarily to chloroplast, chloroplast thylakoid membrane, membrane, biological process and chloroplast thylakoid (*p* < 0.05). In addition, we found genes significantly enriched for photosynthesis (22), cell walls (23), and protein binding (31) (*p* < 0.05). Other genes were associated mainly with the response to the chloroplast stroma (32), chloroplast envelope (40) and starch degradation (13) (*p* < 0.05). The KEGG pathway enrichment analysis showed that 31 DEGs had KEGG pathway annotations (Supplementary Table S5). The significantly enriched pathways (*p* < 0.05) included metabolic pathways, biosynthesis of secondary metabolites, and cysteine and methionine metabolism. Top 20 up- and downregulated genes are listed in Supplementary Table S6, including *O*-fucosyltransferase family protein, glycine/proline-rich protein, photosystem II subunit S, and so on (according to *Arabidopsis* genomic BLAST).

### 3.5. Important Differentially Expressed Genes Responses to Etiolation

Highly dynamic changes in transcript abundance were observed in the etiolation treatment (|log2 (ratio)| ≥ 2; Supplementary Table S7) including unigenes sharing high sequence similarity with genes related to hormone metabolism and response (cytokinin dehydrogenase 1, 1-aminocyclopropane-1-carboxylate oxidase and cytokinin oxidase/dehydrogenase 6); photosynthesis (chlorophyll a-b binding protein 6, photosystem (PS) I type III chlorophyll a/b-binding protein and chlorophyll a-b binding protein CP26), transcription factors (zinc finger protein STZ/ZAT10, NAC transcription factor (*NAM*, *ATAF1/2*, *CUC1/2*), RD26 and dehydration-responsive element-binding protein 1F), signaling pathways (ABC transporter G family member 40, lecithin retinol acyltransferase domain protein and copper-transporting ATPase RAN1), stress and wound respond (heat shock protein 90.1, peroxisomal (S)-2-hydroxy-acid oxidase GLO1, kunitz family trypsin and protease inhibitor protein), starch and sucrose metabolism (fructose-bisphosphate aldolase 1, fructose-1,6-bisphosphatase and beta-amylase). These changes in transcript expression patterns suggested that the mechanism in which etiolation promotes adventitious root formation might be associated with the energy supply, phytohormone synthesis, or signaling pathways.

## 4. Discussion

As early as 1961, Frolich attempted to improve avocado propagation by growing stock plants in darkness, and demonstrated that etiolation improves rooting [22]. Since then, several studies have focused on the mechanisms by which etiolation improves adventitious root formation of stem cuttings. Dai et al. [23] suggested that dark conditions may help tissues maintain a balance between endogenous auxin and cytokinins levels, and that etiolation may stimulate redifferentiation of plant cells and speed up the process of organogenesis. Other researchers have shown that darkness weakens the lignification of shoots, and that they develop less sclerenchyma; thus, there are a fewer barriers to the growth of root primordia [24]. In addition, thinner cell walls and fewer cell wall deposits facilitate penetration of plant growth regulators into cells [24].

The adventitious root formation of tetraploid black locust cuttings was much harder to achieve than that of diploid black locust cuttings and the reduced formation of adventitious roots in tetraploid black locust cuttings remains unclear. In our study, we compared the transcriptomes of E and NE tissues. Transcriptome analysis is important for determining the molecular constituents of cells and tissues and for interpreting the functional elements of the genome. In this study, we assessed differential global gene expression in NE and E juvenile stems, with the aim of discovering genes associated with rooting. We examined the global gene expression of NE and E juvenile stem bases treated with IBA for 1 h. *Glycine max* had the greatest number of hits to contigs from the black locust during transcriptome assembly, indicating high overall sequence similarity. The average length of the transcripts was 755 bp and the N50length (shortest sequence length at 50% of the genome) was 1158 bp, indicating that our contigs were well assembled.

### 4.1. Gene Annotation and Enrichment Analysis of Differential Expressed Genes

Gene annotation and pathway analysis provided an overview of black locust functional regulation under the etiolation at the whole-transcriptome level. In the GO enrichment analysis of all DEGs, chloroplast and chlorophyll-related terms were enriched significantly in the transcriptome data. For example, chlorophyllase, which participates in chlorophyll degradation, was increased significantly in response to etiolation [25]. The loss of the green color in E tissues results from chlorophyll degradation in chloroplasts.

According to the KEGG enrichment analysis, pathways associated with metabolic pathways and the biosynthesis of secondary metabolites were most influenced by etiolation. For example, the 40S ribosomal protein S5A, classified in the ribosome pathway, was expressed only in E tissues. The 40S ribosomal protein S5A is one of two genes encoding the ribosomal protein S5. Weijers found most cell division processes were delayed or disturbed in a heterozygous *Arabidopsis thaliana* mutant, and development was arrested completely at an early embryonic stage in a homozygous mutant [26]. This indicates that cell division and development of E tissues may be postponed or restricted. The results also showed DEGs in phytohormone metabolism and responses, wound and stress responses, as well as starch and sucrose metabolism. In our study, only 31 genes were mapped in KEGG pathways. This may be because there are only a few reports associated to the study of black locust proteins.

### 4.2. Peroxidase and Polyphenol Oxidase Activity Phenylene oxide, Peroxidase and Indole Acetic Acid Oxidase

Peroxidases are major antioxidant enzymes that catalyze the oxidation of a diverse group of organic compounds in plant cells using H_2_O_2_. Peroxidases are involved in scavenging H_2_O_2_ and in plant growth, development, lignification, suberisation, and cross-linking of cell wall compounds [27]. Peroxidases serve as markers for rooting ability in some species; there have been reports of a positive correlation between POD activity and rooting ability [28]. Peroxidase activity in both groups increased during the first five days and then decreased; similar changes have been observed in other plants [29]. Peroxidase activity changed during rooting; indeed, POD activity changes have been proposed as biochemical markers for successive rooting phases. Peroxidase activity in E tissues was enhanced significantly faster than in NE tissues during the first three days. Although excessive H_2_O_2_ accumulation causes lipid peroxidation, membrane damage, and inactivation of enzymes, H_2_O_2_ concentrations at “normal” levels play a role in regulating development and growth. Li et al. [30] suggested that higher concentrations of H_2_O_2_ are required during the formation and development of adventitious roots in cucumber and mung bean. Some studies have also indicated that H_2_O_2_ may function as a signaling molecule involved in the formation and development of adventitious roots. Li et al. [30] suggested that exogenous IBA may induce overproduction of H_2_O_2_ and promote adventitious root formation via a pathway involving H_2_O_2_ in mung bean seedlings. In our study, the transcriptome sequencing results showed that the glycolate oxidase 1 gene significantly increased in E tissues. Overexpressed glycolate oxidase 1 in chloroplasts causes the accumulation of H_2_O_2_ in *A. thaliana* [31]. However, we also found some genes associated with decomposing peroxides, in particular H_2_O_2_ also significantly increased in E tissues such as PODs 51, 53, and 54 as well as peroxiredoxin Q. These results suggest that E tissues may first increase the production of H_2_O_2_, after which excessive H_2_O_2_ is degraded by increasing POD production so that H_2_O_2_ content is maintained at a normal level, promoting adventitious root formation.

### 4.3. Differential Expressed Genes Related to Phytohormone Generation and Responses

Cutting the mother plant leads to numerous stressors due to interruption of water and nutrient supplies, altered transport of phytohormones, and activation of wound responses [32]. The formation of adventitious roots is a complex process that involves successive developmental phases, requiring different hormonal signals and other endogenous factors. Hormones such as auxin, jasmonic acid (JA), ethylene, and GA are involved in adventitious root formation. Wounding from cutting would be expected to stimulate the expression of JA biosynthetic genes, thereby increasing JA levels. In our study, some genes associated with JA synthesis were more highly expressed in E tissues such as 12-oxophytodienoate reductase 2 and lipoxygenase 2. 12-Oxophytodienoate reductase 2 encodes a member of a α/β-barrel-fold family of flavin mononucleotide (FMN)-containing oxidoreductases that is predominantly expressed in roots and is upregulated by JA and salicylic acid. Tani et al. [33] showed that 12-oxophytodienoate reductase is involved in the biosynthesis of JA in rice. Lipoxygenase is also required for wound-induced JA accumulation. By studying a 9-hydroxyoctadecatrienoic acid-insensitive mutant, Vellosillo et al. [34] showed that oxylipins produced by the 9-lipoxygenase pathway regulate lateral root development in *Arabidopsis*. However, Da Costa et al. [35] suggested that JA might have dual functions as an inducer of sink establishment in the rooting zone and as a negative regulator of root initiation.

Ethylene plays a positive role in adventitious root formation by modulating auxin transport as a central point in ethylene–auxin crosstalk. Ethylene induces epidermal cell death at the site of adventitious root emergence and accelerated root growth [36]. In our study, we found that some genes involved in ethylene biosynthetic processes were significantly expressed in E tissues such as 1-aminocyclopropane-1-carboxylate oxidase [37]. Ethylene is required for chlorophyll degradation in the peels of two citrus species [38], indicating that etiolation may increase ethylene in tissues. Auxin positively regulates adventitious root formation in most plant species. Elevated endogenous or exogenous concentrations of auxin increase the formation of adventitious roots [39,40,41]. Gutierrez et al. [42] reported that auxin controls adventitious root initiation by regulating JA homeostasis. Reductions in auxin signaling or transport, due to mutations or inhibitors, reduce both adventitious root initiation and elongation [43,44]. In our study, increased IAA content in the stem basal end of E cuttings, as well as certain genes involved in auxin transport, such as auxin efflux carrier family protein, auxin efflux carrier-like protein, chalcone, and stilbene synthase family proteins, were detected. Ludwig-Müller et al. [45] suggested that adventitious rooting in stem segments may be due to an interaction between endogenous IAA and exogenous IBA. Residual endogenous IAA is transported to the basal end of segments in stem explants, inducing root formation. Our results suggest that etiolation may reinforce auxin polar transport and further stimulate adventitious root formation in stems. We also found some auxin-responsive genes such as thermospermine synthase ACAULIS5 and root meristem growth factor 9. ACAULIS5 participates in the synthesis of thermospermine, and is an isomer of spermidine that is widely distributed throughout plants but has not been detected in mammalian cells [46]. ACAULIS5 controls xylem differentiation and vascular development [47]. Using histochemical β-glucuronidase (GUS) staining, Vera-Sirera et al. [48] showed that ACAULIS5 promotes the formation of adventitious roots; this was reinforced by increased IAA levels in the hypocotyl. Root meristem growth factor 9 encodes a root growth factor (RGF) required for maintenance of the root stem cell niche and amplifying cell proliferation [49]. Some studies have suggested that RGFs function in regulating the direction of root growth and lateral root development [50]. However, we found that auxin response factor 6 was significantly downregulated, which is a positive regulator of adventitious rooting. Light promotes expression of auxin response factor 6; thus, the downregulation of auxin response factor 6 in E tissues may be due to the lack of light [51].

Gibberellin content in E tissues was significantly higher than that in the control 0 d cuttings. Mauriat et al. [52] described how GA3s affects auxin transport and thus inhibits adventitious rooting. However, Niu et al. [53] showed that an appropriate level of GA3 in vascular tissues is necessary for regulating adventitious root development. Da Costa et al. [35] suggested that GA may have a phase-dependent effect, inhibiting root induction but stimulating formation. In this study, we found that GA_3_-regulated protein 14 was significantly expressed in E tissues. Sun et al. [54] showed that GA_3_-regulated protein 14 is expressed in the elongation zone of roots and suggested that it functions in promoting cell elongation. However, it remains unknown whether this gene is associated with adventitious root formation.

### 4.4. Differential Expressed Genes Related to Starch and Sucrose Metabolism

A considerable number of transcripts differentially expressed following etiolation treatment encoded putative genes related to starch and sucrose metabolism such as phosphoglucomutase 3, beta-amylase, and fructose-1,6-bisphosphatase. Starch is an important source of carbohydrates, providing energy for the initiation and development of root primordia in woody cuttings [55]. Auxins promote starch hydrolysis and mobilize sugars and nutrients to the cutting base [56]. β-Amylases participate in starch degradation [57] and a reduction of phosphoglucomutase 3 in *Arabidopsis* decreases starch content, but increases sucrose, maltose and cell wall components [53], suggesting that E cuttings obtained more energy from starch than NE cuttings. In our study, we also found that fructose-1,6-bisphosphatase participates in sucrose biosynthesis and increases significantly in etiolated tissues [58]. Our results indicate that more sucrose may accumulate in etiolated tissues, possibly promoting adventitious root formation.

### 4.5. Differentially Expressed Genes Related to Stress and Wound Responses

In our study, genes encoding proteins involved in stress and wound responses such as 12-oxophytodienoate reductase 2, Kunitz family trypsin and protease inhibitor protein and chitinase-like protein were differentially expressed in E tissues [59,60]. 12-Oxophytodienoate reductase 2 encodes a FMN-containing oxidoreductase that is mainly expressed in roots and is upregulated by JA and salicylic acid [61]. Tani et al. [33] showed that 12-oxophytodienoate reductase is involved in the synthesis of JA in rice, which is associated with adventitious root formation. Kunitz family trypsin and protease inhibitor proteins play important roles in plant defenses against wounds and stressors; they also respond to high light intensity [59]. By identifying the protein dynamics of E *Arabidopsis* hypocotyls, Irshad et al. [62] showed the expression of inhibitor family I3 (Kunitz-P family) proteins in elongating cells, indicating that Kunitz family trypsin and protease inhibitor proteins may participate in cell elongation in E tissues. Chitinase is expressed exclusively under environmental stress conditions and has been implicated in defense responses against pathogens. Through a proteomics approach, Kwon et al. [63] showed that chitinase may be involved in the structural organisation of cell walls and may participate in an active defense system in *A. thaliana*. In addition to active chitinases, some plants also express chitinase-like proteins, which appear to lack chitinolytic activity. For example, Zhang et al. [64] reported that two homologous cotton chitinase-like proteins (*GhCTL*1 and *GhCTL*2) are preferentially expressed during secondary cell wall deposition in cotton fibre cells, and are responsible for cellulose biosynthesis during primary cell wall formation and cellulose biosynthesis during secondary cell wall formation in vascular tissues of *A. thaliana*. Using a chitinase-like protein-encoding *AtCTL*2 gene mutant, Hossain et al. [65] suggested that *AtCTL*2 is required for appropriate cell wall biosynthesis in *Arabidopsis* seedlings. In our study, we found a significant decrease in chitinase-like protein 2 in E tissues, which may negatively regulate cell wall formation and promote adventitious root formation. Proline is an important osmoprotectant when resisting stress, and has also been proposed to supply energy [66]. In our study, higher levels of proline dehydrogenase (PDH) 2 and proline transporter 1 were expressed in E tissues. Proline dehydrogenase catalyses the first and rate-limiting step in the proline catabolic pathway and is involved in proline degradation [67]. When cells are released from stress, proline is oxidized to glutamate by PDH2 and pyrroline-5-carboxylate dehydrogenase [68]. Funck et al. [69] showed that PDH2 overexpression enables utilization of proline as the sole nitrogen source for growth, and hypothesized that it plays an important role in proline homeostasis in the vasculature, particularly under stress conditions that promote proline accumulation in *Arabidopsis*. Schwambach et al. [70] demonstrated that adventitious root numbers and length were significantly affected by the nitrogen source in *Eucalyptus globulus* microcuttings. Similar conclusions were reached regarding cultured *Petunia hybrida* and *Euphorbia pulcherrima* [71,72]. These studies indicate that an appropriate increase in nitrogen sources may promote the rooting of cuttings. Etiolation may increase nitrogen content by enhancing proline accumulation and degradation in the stem base, thereby promoting adventitious root formation.

### 4.6. Other Transcripts with Highly Differential Expression

Differential expressed transcripts that accumulated to high levels in the NE or E libraries revealed key genes potentially related to adventitious root formation, such as Clavata3/emobryo-surrounding region (CLE)-like peptides, root hair defective 3 GTP-binding protein (RHD3), translation initiation factor eIF-5A, and translationally controlled tumor protein (TCTP). The CLE-like peptides control the pattern of root growth and lateral root development in Arabidopsis and are expressed primarily in the stem cell area and the innermost layer of central columella cells [50,73]. The RHD3 appears to be required for root hair development [74]. We also identified a significant increase in translation initiation factor eIF-5A. The eIF5A is essential for cell proliferation and viability. Belda-Palazón et al. [75] showed that spermidine-mediated activation of eIF5A by hypusination is involved in several aspects of plant biology, such as the control of root architecture and root hair growth. The TCTP serves as guanine nucleotide exchange factor in the target of rapamycin (TOR) signaling pathway and is expressed throughout the plant, with the highest levels seen in the meristematic regions of the shoot and root. Plants lacking TCTP develop defects in lateral and primary root growth and in root hair growth. Toscano-Morales et al. [76] suggested that long-distance delivery of the TCTP protein and messenger RNA (mRNA) is required for the induction of adventitious roots. We also found that some genes associated with the lignin biosynthetic process were highly expressed in E tissues, such as cinnamoyl-coenzyme A (coA) reductase and cinnamyl alcohol dehydrogenase 6 [77,78]. Quan et al. [79] reported that tetraploid black locust cuttings with 19.5% lignin had a significantly higher rooting rate (60.4%) than those with only 10.6% lignin (12.9%), indicating that an appropriately higher lignin content in cuttings may be better for adventitious root formation.

In this study, a general understanding of the transcriptomic response of tetraploid black locust to etiolation treatment and representative genes involved in the response were presented, based on annotation and enrichment analyses. The information generated provides a foundation for additional studies on the genetic response to etiolation and adventitious root formation in tetraploid black locust and other tree species. Given that the whole-genome sequence of black locust has not yet been elucidated, our study provides reference data for molecular biology research on the black locust. However, many details of the molecular mechanism(s) by which etiolation promotes adventitious root formation in the tetraploid black locust remain unclear. These will be our research focus in future studies.

## Supplementary Materials

The following are available online at www.mdpi.com/2073-4425/8/12/345/s1. Table S1: List of the experimental individuals of *R. pseudoacacia* L., Table S2: Summary of the tetraploid *R. pseudoacacia* transcriptome, Table S3: Categorization of tetraploid *R. pseudoacacia* with KEGG biochemical pathways, Table S4: The GO analysis of the DETs, Table S5: The KEGG analysis of the DETs, Table S6: Top 20 up- and downregulated genes in response to etiolation, Table S7: Transcript expression level of part differentially expressed genes.
