# Supplementary material for: Characterization of the Transcriptome and Gene Expression of Tetraploid Black Locust Cuttings in Response to Etiolation
Source: Genes (Basel). 2017 Nov 24;8(12):345. doi: 10.3390/genes8120345 (PMC5748663; doi:10.3390/genes8120345)
Supplement: Supplementary file 1 [file genes-08-00345-s001.zip › genes-231221_supplementary_final/genes-231221_final_supplementary.docx]

Supplementary Materials: Characterization of the Transcriptome and Gene Expression of Tetraploid Black Locust Cuttings in Response to Etiolation

Nan Lu ^1,2,†^, Li Dai ^1,†^, Zijing Luo ^1^, Shaoming Wang ^3^, Yanzhong Wen ^4^, Hongjing Duan ^1^, Rongxuan Hou ^1^, Yuhan Sun ^1^, and Yun Li ^1,^*

^1^ Beijing Advanced Innovation Center for Tree Breeding by Molecular Design, National Engineering Laboratory for Tree Breeding, Key Laboratory of Genetics and Breeding in Forest Trees and Ornamental Plants of Ministry of Education, College of Biological Sciences and Technology, Beijing Forestry University, Beijing 100083, China; [ln_890110@163.com](mailto:ln_890110@163.com) (N.L.); [daili.jiayou@163.com](mailto:daili.jiayou@163.com) (L.D.); [luozijingbjfu@163.com](mailto:luozijingbjfu@163.com) (Z.L.); [duan673356712@126.com](mailto:duan673356712@126.com) (H.D.); [hrxvshzy@163.com](mailto:hrxvshzy@163.com) (R.H.); [syh831008@163.com](mailto:syh831008@163.com) (Y.S.)

^2^ State Key Laboratory of Forest Genetics and Tree Breeding, Key Laboratory of Tree Breeding and Cultivation of State Forestry Administration, Research Institute of Forestry, Chinese Academy of Forestry, Beijing 100091, China

^3^ The State-Owned Luoning County Lv Cun Forest Farm, Luoning 471711, China; [lclc873@163.com](mailto:lclc873@163.com)

^4^ The State-Owned Ji County Nursery of Forests, Linfen 042200, China; [jxzzywyz@163.com](mailto:jxzzywyz@163.com).

***** Correspondence: yunli@bjfu.edu.cn; Tel.: +86-10-6233-6094

† These authors contributed equally to this work.

**Table S1.** Summary of sequencing and assembly.

| **Sample ID** | **Replicates** | **Raw read** | **Quality trimmed** | **Adaptor trimmed** | **rRNA** **trimmed** | **Clean ratio** |
| --- | --- | --- | --- | --- | --- | --- |
| Non-etiolated treatment (control) | 1 | 62,315,544 | 61,876,804 | 60,479,091 | 59,050,378 | 94.76% |
|  | 2 | 87,977,154 | 87,501,884 | 85,534,080 | 83,700,310 | 95.14% |
|  | 3 | 52,642,822 | 52,318,969 | 50,943,879 | 49,830,450 | 94.66% |
| Average |  | 67,645,173 | 67,232,552 | 65,652,350 | 64,193,713 | 94.85% |
| Etiolated treatment | 1 | 62,385,126 | 62,060,405 | 60,612,441 | 59,321,960 | 95.09% |
|  | 2 | 61,606,896 | 55,662,423 | 31,551,135 | 29,184,730 | 47.37% |
|  | 3 | 61,420,516 | 60,795,194 | 58,393,997 | 56,877,804 | 92.60% |
| Average |  | 61,804,179 | 59,506,007 | 50,185,858 | 48,461,498 | 78.35% |

**Table S2.** Summary of the tetraploid *R. pseudoacacia* transcriptome.

| **Statistic** | **Count** | **Total length (bp)** | **N25 (bp)** | **N50 (bp)** | **N75 (bp)** | **Average length** | **Longest (bp)** | **N%** | **GC%** | **Annotation count** | **Annotation rate** |
| --- | --- | --- | --- | --- | --- | --- | --- | --- | --- | --- | --- |
| Contigs | 113,233 | 78,059,940 | 2,015 | 1,030 | 474 | 689 | 15,626 | 0.4 | 37.6 | - | - |
| Primary UniGene | 126,350 | 77,825,120 | 1,735 | 880 | 424 | 616 | 15,626 | 0.4 | 37.6 | - | - |
| Final UniGene | 107,564 | 81,197,064 | 2,221 | 1,158 | 536 | 755 | 16,999 | 3.84 | 36.28 | 52,590 | 48.89% |

**Table S3.** Categorization of tetraploid *R. pseudoacacia* with KEGG biochemical pathways.

| **Pathway ID** | **KEGG categories** | **Mapped_KO** | **Unigene no.** | **Ratio of no. (%)** |
| --- | --- | --- | --- | --- |
| ko01100 | Metabolic pathways | 454 | 681 | 2.35 |
| ko01110 | Biosynthesis of secondary metabolites | 230 | 376 | 1.30 |
| ko01130 | Biosynthesis of antibiotics | 109 | 169 | 0.58 |
| ko01120 | Microbial metabolism in diverse environments | 88 | 155 | 0.53 |
| ko04141 | Protein processing in endoplasmic reticulum | 40 | 114 | 0.39 |
| ko01200 | Carbon metabolism | 63 | 106 | 0.37 |
| ko00500 | Starch and sucrose metabolism | 31 | 105 | 0.36 |
| ko01230 | Biosynthesis of amino acids | 62 | 102 | 0.35 |
| ko05169 | Epstein-Barr virus infection | 30 | 87 | 0.30 |
| ko03010 | Ribosome | 57 | 80 | 0.28 |
| ko00620 | Pyruvate metabolism | 24 | 77 | 0.27 |
| ko00190 | Oxidative phosphorylation | 43 | 68 | 0.23 |
| ko00230 | Purine metabolism | 45 | 67 | 0.23 |
| ko00010 | Glycolysis / Gluconeogenesis | 28 | 63 | 0.22 |
| ko04144 | Endocytosis | 27 | 55 | 0.19 |
| ko03013 | RNA transport | 42 | 54 | 0.19 |
| ko00195 | Photosynthesis | 15 | 52 | 0.18 |
| ko04075 | Plant hormone signal transduction | 24 | 51 | 0.18 |
| ko00940 | Phenylpropanoid biosynthesis | 15 | 47 | 0.16 |
| ko00240 | Pyrimidine metabolism | 34 | 46 | 0.16 |
| ko03018 | RNA degradation | 30 | 43 | 0.15 |
| ko03015 | mRNA surveillance pathway | 30 | 42 | 0.14 |
| ko05230 | Central carbon metabolism in cancer | 11 | 42 | 0.14 |
| ko04145 | Phagosome | 18 | 41 | 0.14 |
| ko04151 | PI3K-Akt signalling pathway | 22 | 40 | 0.14 |
| ko03040 | Spliceosome | 36 | 39 | 0.13 |
| ko05203 | Viral carcinogenesis | 24 | 39 | 0.13 |
|  | Others |  | 3408 | 11.75 |

Ratio of no.: number of unigenes/total genes in all mapped KO pathways.

**Table S7.** Transcript expression level of part differentially expressed genes.

| **Unigene id** | **Unigene_length** | **Ath**  **BLAST** | **E**  **RPKM** | **NE**  **RPKM** | **UP/DOWN regulated** | ***p*-value** |
| --- | --- | --- | --- | --- | --- | --- |
| Hormone metabolism and response | | | | | | |
| Nonyellowing2_no_rRNA.1_(paired)_contig_3822 | 2796 | Thermospermine synthase ACAULIS5 | 25.01667 | 3.188976 | UP | 2.38E-05 |
| Nonyellowing2_no_rRNA.1_(paired)_contig_57734 | 268 | lecithin retinol acyltransferase domain protein | 0.543333 | 14.55347 | DOWN | 5.58E-04 |
| First_Contig51 | 4191 | auxin response factor 6 | 0.026667 | 14.77261 | DOWN | 5.58E-04 |
| Nonyellowing2_no_rRNA.1_(paired)_contig_13600 | 2347 | cytokinin oxidase/dehydrogenase 6 | 9.7 | 51.71855 | DOWN | 1.36E-07 |
| Nonyellowing2_no_rRNA.1_(paired)_contig_41663 | 1921 | cytokinin dehydrogenase 1 | 1.236667 | 17.3687 | DOWN | 4.43E-04 |
| Nonyellowing2_no_rRNA.1_(paired)_contig_78841 | 2164 | auxin efflux carrier-like protein | 21.46333 | 0.129654 | UP | 1.78E-06 |
| Nonyellowing2_no_rRNA.1_(paired)_contig_3822 | 2796 | Thermospermine synthase ACAULIS5 | 25.01667 | 3.188976 | UP | 2.38E-05 |
| Nonyellowing2_no_rRNA.1_(paired)_contig_8602 | 1772 | IAA-amino acid hydrolase IAR3 | 51.61333 | 5.692749 | UP | 4.74E-11 |
| Nonyellowing2_no_rRNA.1_(paired)_contig_22193 | 246 | 1-aminocyclopropane-1-carboxylate oxidase | 111.7833 | 21.01783 | UP | 4.38E-17 |
| Nonyellowing2_no_rRNA.1_(paired)_contig_18123 | 381 | 1-aminocyclopropane-1-carboxylate oxidase | 70.42333 | 15.20754 | UP | 2.37E-10 |
| Nonyellowing2_no_rRNA.1_(paired)_contig_8411 | 966 | gibberellin-regulated protein 14 | 30.49 | 1.668845 | UP | 3.75E-08 |
| Photosynthesis | | | | | | |
| Nonyellowing2_no_rRNA.1_(paired)_contig_29372 | 457 | light harvesting complex photosystem II subunit 6 | 37.14333 | 0.163333 | UP | 3.02E-11 |
| Nonyellowing2_no_rRNA.1_(paired)_contig_21389 | 1687 | glyceraldehyde 3-phosphate dehydrogenase GAPA2 | 31.43667 | 0.276659 | UP | 1.90E-09 |
| Nonyellowing2_no_rRNA.1_(paired)_contig_22922 | 961 | light-harvesting chlorophyll B-binding protein 3 | 17.60333 | 0.156667 | UP | 2.64E-05 |
| Nonyellowing2_no_rRNA.1_(paired)_contig_22921 | 1023 | light-harvesting chlorophyll B-binding protein 3 | 60.89667 | 0.583622 | UP | 3.63E-18 |
| Nonyellowing2_no_rRNA.1_(paired)_contig_5508 | 1203 | fructose-bisphosphate aldolase 1 | 124.3233 | 1.256722 | UP | 1.53E-36 |
| Nonyellowing2_no_rRNA.1_(paired)_contig_4294 | 1451 | ribulose bisphosphate carboxylase/oxygenase activase | 42.25 | 0.45207 | UP | 9.49E-13 |
| Nonyellowing2_no_rRNA.1_(paired)_contig_667 | 1150 | chlorophyll a-b binding protein 6 | 286.44 | 3.731085 | UP | 1.99E-83 |
| Nonyellowing2_no_rRNA.1_(paired)_contig_23633 | 2266 | thylakoid rhodanese-like protein | 49.26333 | 0.680224 | UP | 7.41E-15 |
| Nonyellowing2_no_rRNA.1_(paired)_contig_828 | 982 | chlorophyll a-b binding protein CP26 | 263.95 | 4.377162 | UP | 6.77E-75 |
| Nonyellowing2_no_rRNA.1_(paired)_contig_4273 | 906 | ribulose bisphosphate carboxylase/oxygenase activase | 111.56 | 1.911763 | UP | 1.40E-32 |
| Nonyellowing2_no_rRNA.1_(paired)_contig_34263 | 1487 | chloroplast stem-loop binding protein | 14.04 | 0.322847 | UP | 3.02E-11 |
| Nonyellowing2_no_rRNA.1_(paired)_contig_6381 | 457 | light harvesting complex photosystem II subunit 6 | 112.67 | 4.431623 | UP | 1.90E-09 |
| Nonyellowing2_no_rRNA.1_(paired)_contig_6226 | 879 | PSI type III chlorophyll a/b-binding protein | 300.1233 | 12.82617 | UP | 2.64E-05 |
| Nonyellowing2_no_rRNA.1_(paired)_contig_5239 | 834 | photosystem I reaction center subunit D-2 | 137.3167 | 6.270678 | UP | 3.63E-18 |
| Nonyellowing2_no_rRNA.1_(paired)_contig_5511 | 1756 | phosphoribulokinase | 73.8 | 3.535588 | UP | 1.53E-36 |
| Nonyellowing2_no_rRNA.1_(paired)_contig_8339 | 4829 | magnesium chelatase subunit H | 40.86667 | 2.952852 | UP | 9.49E-13 |
| Nonyellowing2_no_rRNA.1_(paired)_contig_2104 | 911 | ferredoxin-NADP(+)-oxidoreductase 1 | 65.95 | 5.616228 | UP | 1.99E-83 |
| Nonyellowing2_no_rRNA.1_(paired)_contig_9400 | 784 | photosystem II subunit P-1 | 202.33 | 17.82207 | UP | 7.41E-15 |
| Nonyellowing2_no_rRNA.1_(paired)_contig_4324 | 1411 | photosystem II subunit Q-2 | 77.91 | 7.078999 | UP | 6.77E-75 |
| Nonyellowing2_no_rRNA.1_(paired)_contig_5684 | 1096 | photosystem I subunit F | 158.7967 | 14.55142 | UP | 1.40E-32 |
| Nonyellowing2_no_rRNA.1_(paired)_contig_1769 | 1140 | ferretin 1 | 461.2533 | 43.42731 | UP | 1.96E-04 |
| Nonyellowing2_no_rRNA.1_(paired)_contig_13245 | 942 | plastid-lipid associated protein PAP / fibrillin family protein | 35.31 | 4.049767 | UP | 4.29E-29 |
| Nonyellowing2_no_rRNA.1_(paired)_contig_24893 | 1407 | protein high chlorophyll fluorescence 101 | 15.28333 | 1.886304 | UP | 2.53E-76 |
| Nonyellowing2_no_rRNA.1_(paired)_contig_8193 | 939 | chlorophyllase 2 | 23.87 | 3.533976 | UP | 1.38E-34 |
| Nonyellowing2_no_rRNA.1_(paired)_contig_15397 | 439 | photosystem II reaction center W protein | 36.88 | 5.648376 | UP | 7.27E-19 |
| Nonyellowing2_no_rRNA.1_(paired)_contig_14744 | 272 | photosystem II subunit P-1 | 49.57 | 7.86122 | UP | 4.41E-10 |
| Nonyellowing2_no_rRNA.1_(paired)_contig_1758 | 1182 | oxygen-evolving enhancer protein 1-2 | 388.25 | 62.61781 | UP | 7.67E-15 |
| Nonyellowing2_no_rRNA.1_(paired)_contig_3923 | 811 | photosystem I reaction center subunit N | 230.05 | 39.72191 | UP | 2.01E-43 |
| Transcription factor | | | | | | |
| Nonyellowing2_no_rRNA.1_(paired)_contig_1254 | 1626 | CCCH-type zinc finger protein SOMNUS | 32.56 | 0.642097 | UP | 9.53E-10 |
| Nonyellowing2_no_rRNA.1_(paired)_contig_35288 | 818 | NAC domain containing protein 80 | 15.11 | 1.200759 | UP | 5.47E-04 |
| Nonyellowing2_no_rRNA.1_(paired)_contig_8331 | 1158 | zinc finger protein STZ/ZAT10 | 151.8733 | 19.73202 | UP | 3.98E-28 |
| Nonyellowing2_no_rRNA.1_(paired)_contig_9996 | 1400 | dehydration-responsive element-binding protein 1F | 21.19333 | 3.187769 | UP | 2.34E-04 |
| Nonyellowing2_no_rRNA.1_(paired)_contig_10146 | 1389 | Putative GATA transcription factor 22 | 53.59667 | 9.966322 | UP | 3.80E-09 |
| Signaling passway | | | | | | |
| Nonyellowing2_no_rRNA.1_(paired)_contig_12304 | 1365 | protein NDR1/HIN1-like 10 | 102.3667 | 13.45648 | UP | 3.19E-19 |
| Nonyellowing2_no_rRNA.1_(paired)_contig_30473 | 1200 | aluminum induced protein with YGL and LRDR motifs | 278.06 | 37.28318 | UP | 1.25E-49 |
| Nonyellowing2_no_rRNA.1_(paired)_contig_1729 | 2061 | cytochrome b6-f complex iron-sulfur subunit | 30.45 | 4.34088 | UP | 5.14E-06 |
| Nonyellowing2_no_rRNA.1_(paired)_contig_31067 | 200 | ABC transporter G family member 40 | 4.7 | 25.99194 | DOWN | 2.27E-04 |
| Nonyellowing2_no_rRNA.1_(paired)_contig_34557 | 918 | sigma factor binding protein 1 | 31.80333 | 7.558408 | UP | 5.36E-05 |
| Nonyellowing2_no_rRNA.1_(paired)_contig_22729 | 1420 | LRR receptor-like serine/threonine-protein kinase GSO1 | 7.016667 | 39.09963 | DOWN | 7.22E-06 |
| Nonyellowing2_no_rRNA.1_(paired)_contig_3532 | 1008 | uncharacterized protein | 7.77 | 44.01009 | DOWN | 5.54E-07 |
| Nonyellowing2_no_rRNA.1_(paired)_contig_34885 | 980 | translationally-controlled tumor protein-like protein | 15.78 | 97.07866 | DOWN | 9.83E-15 |
| Nonyellowing2_no_rRNA.1_(paired)_contig_57734 | 268 | lecithin retinol acyltransferase domain protein | 0.543333 | 14.55347 | DOWN | 5.58E-04 |
| First_Contig21 | 3419 | CHY and CTCHY and RING-type zinc finger protein | 5.623333 | 300.472 | DOWN | 1.88E-77 |
| First_Contig421 | 839 | leucine-rich receptor-like protein kinase | 0.256667 | 15.52535 | DOWN | 2.99E-04 |
| Nonyellowing2_no_rRNA.1_(paired)_contig_42991 | 2312 | flavin-dependent monooxygenase 1 | 0.14 | 47.03213 | DOWN | 3.58E-13 |
| Nonyellowing2_no_rRNA.1_(paired)_contig_11601 | 1111 | L-type lectin-domain containing receptor kinase IX.1 | 0.876667 | 667.9659 | DOWN | 1.59E-04 |
| First_Contig628 | 218 | copper-transporting ATPase RAN1 | 0 | 16.50114 | DOWN | - |
| First_Contig3256 | 975 | uncharacterized protein | 0 | 372.0184 | DOWN | - |
| Stress and wound respond | | | | | | |
| Nonyellowing2_no_rRNA.1_(paired)_contig_21346 | 971 | 12-oxophytodienoate reductase 2 | 43.41 | 1.561867 | UP | 6.83E-12 |
| Nonyellowing2_no_rRNA.1_(paired)_contig_6523 | 1614 | proline dehydrogenase 2 | 49.77 | 10.86958 | UP | 1.03E-07 |
| Nonyellowing2_no_rRNA.1_(paired)_contig_6099 | 1023 | 12-oxophytodienoate reductase 2 | 83.81 | 20.82769 | UP | 4.94E-11 |
| Nonyellowing2_no_rRNA.1_(paired)_contig_6764 | 334 | probable aldo-keto reductase 4 | 25.99 | 107.9171 | DOWN | 3.63E-12 |
| Nonyellowing2_no_rRNA.1_(paired)_contig_10180 | 657 | chitinase-like protein 2 | 29.73333 | 146.4826 | DOWN | 2.18E-18 |
| Nonyellowing2_no_rRNA.1_(paired)_contig_5106 | 578 | lipoxygenase 1 | 114.31 | 607.7471 | DOWN | 6.23E-76 |
| Nonyellowing2_no_rRNA.1_(paired)_contig_75978 | 1853 | trans-cinnamate 4-monooxygenase | 3.963333 | 23.59666 | DOWN | 2.09E-04 |
| Nonyellowing2_no_rRNA.1_(paired)_contig_52645 | 2000 | cytochrome P450, family 711, subfamily A, polypeptide 1 | 9.2 | 62.09315 | DOWN | 4.49E-10 |
| Nonyellowing2_no_rRNA.1_(paired)_contig_12914 | 1787 | lipoxygenase 1 | 14.31333 | 103.534 | DOWN | 1.13E-16 |
| First_Contig24 | 2084 | lipoxygenase 1 | 0.036667 | 15.64174 | DOWN | 2.99E-04 |
| Nonyellowing2_no_rRNA.1_(paired)_contig_17233 | 3172 | lipoxygenase 2 | 15.21 | 0.663333 | UP | 1.01E-04 |
| Nonyellowing2_no_rRNA.1_(paired)_contig_27964 | 1908 | glutamine synthetase 2 | 37.27667 | 3.521123 | UP | 1.82E-08 |
| Nonyellowing2_no_rRNA.1_(paired)_contig_75389 | 292 | heat shock protein 90.1 | 0.483333 | 23.0682 | DOWN | 1.88E-06 |
| Nonyellowing2_no_rRNA.1_(paired)_contig_23152 | 205 | heat shock protein 70-4 | 7.783333 | 39.37533 | DOWN | 7.22E-06 |
| First_Contig723 | 249 | heat shock protein 70-4 | 1.00E-05 | 14.16462 | DOWN | 0.000558 |
| Nonyellowing2_no_rRNA.1_(paired)_contig_42123 | 917 | heat shock cognate protein 70-1 | 15.72 | 71.15462 | DOWN | 5.12E-09 |
| Nonyellowing2_no_rRNA.1_(paired)_contig_97425 | 262 | heat shock cognate protein 70-1 | 1.38 | 64.69076 | DOWN | 1.49E-16 |
| Nonyellowing2_no_rRNA.1_(paired)_contig_79487 | 517 | heat shock cognate protein 70-1 | 0.066667 | 67.1257 | DOWN | 1.40E-18 |
| Nonyellowing2_no_rRNA.1_(paired)_contig_88924 | 390 | heat shock cognate protein 70-1 | 1.00E-05 | 23.09532 | DOWN | 1.88E-06 |
| Nonyellowing2_no_rRNA.1_(paired)_contig_10854 | 1628 | peroxisomal (S)-2-hydroxy-acid oxidase GLO1 | 93.22 | 0.55 | UP | 1.61E-28 |
| Nonyellowing2_no_rRNA.1_(paired)_contig_30394 | 1166 | peroxiredoxin Q | 14.53 | 0.334102 | UP | 0.000196 |
| Nonyellowing2_no_rRNA.1_(paired)_contig_11805 | 614 | peroxidase 54 | 226.29 | 44.06339 | UP | 6.48E-33 |
| Nonyellowing2_no_rRNA.1_(paired)_contig_8151 | 1119 | pathogenesis-related thaumatin-like protein | 46.63333 | 11.50389 | UP | 1.31E-06 |
| Nonyellowing2_no_rRNA.1_(paired)_contig_90435 | 1220 | pathogenesis-related protein 1 | 107.3333 | 9.162172 | UP | 2.15E-23 |
| Nonyellowing2_no_rRNA.1_(paired)_contig_60621 | 249 | kunitz family trypsin and protease inhibitor protein | 3.083333 | 39.42479 | DOWN | 2.66E-08 |
| Nonyellowing2_no_rRNA.1_(paired)_contig_21986 | 2600 | glycosyl hydrolase 9C2 | 32.69333 | 6.93974 | UP | 1.90E-05 |
| Nonyellowing2_no_rRNA.1_(paired)_contig_11889 | 750 | glycine/proline-rich protein | 15.43 | 0.06 | UP | 0.000101 |
| Nonyellowing2_no_rRNA.1_(paired)_contig_103 | 781 | dehydration-responsive protein RD22 | 54.11333 | 252.4693 | DOWN | 3.10E-29 |
| Nonyellowing2_no_rRNA.1_(paired)_contig_33260 | 803 | dehydration-responsive protein RD22 | 1.126667 | 78.45325 | DOWN | 1.62E-20 |
| Nonyellowing2_no_rRNA.1_(paired)_contig_90428 | 257 | dehydration-responsive protein RD22 | 1.00E-05 | 35.37543 | DOWN | 8.43E-10 |
| Nonyellowing2_no_rRNA.1_(paired)_contig_10180 | 657 | chitinase-like protein 2 | 29.73333 | 146.4826 | DOWN | 2.18E-18 |
| Nonyellowing2_no_rRNA.1_(paired)_contig_5188 | 208 | chitinase A | 2059.44 | 81.44482 | UP | 0 |
| Nonyellowing2_no_rRNA.1_(paired)_contig_22292 | 210 | chitinase A | 1436.877 | 66.10533 | UP | 0 |
| Nonyellowing2_no_rRNA.1_(paired)_contig_7480 | 764 | chitinase A | 421.1733 | 19.90756 | UP | - |
| Nonyellowing2_no_rRNA.1_(paired)_contig_9996 | 1400 | dehydration-responsive element-binding protein 1F | 21.19333 | 3.187769 | UP | 2.34E-04 |
| Nonyellowing2_no_rRNA.1_(paired)_contig_27366 | 1737 | aspartyl protease-like protein | 252.3033 | 18.01838 | UP | 1.25E-56 |
| Nonyellowing2_no_rRNA.1_(paired)_contig_92695 | 1238 | aquaporin PIP2-1 | 0.676667 | 53.957 | DOWN | 7.27E-15 |
| Nonyellowing2_no_rRNA.1_(paired)_contig_23084 | 887 | ACT domain-containing protein | 53.10667 | 13.22127 | UP | 2.76E-07 |
| Nonyellowing2_no_rRNA.1_(paired)_contig_41600 | 3043 | ABC transporter G family member 11 | 40.97 | 4.214464 | UP | 1.51E-08 |
|  | | | | | | |
| Nonyellowing2_no_rRNA.1_(paired)_contig_7907 | 1274 | fructose-1,6-bisphosphatase | 60.11 | 3.616089 | UP | 4.03E-15 |
| Nonyellowing2_no_rRNA.1_(paired)_contig_58260 | 687 | sugar transport protein 13 | 40.24333 | 6.844802 | UP | 1.34E-07 |
| Nonyellowing2_no_rRNA.1_(paired)_contig_57870 | 875 | beta-amylase | 20.04 | 2.282414 | UP | 1.13E-04 |
| Nonyellowing2_no_rRNA.1_(paired)_contig_5508 | 1203 | fructose-bisphosphate aldolase 1 | 124.3233 | 1.256722 | UP | 1.53E-36 |
| Nonyellowing2_no_rRNA.1_(paired)_contig_5497 | 1956 | phosphoglycerate kinase | 124.87 | 23.46668 | UP | 3.99E-19 |
| Nonyellowing2_no_rRNA.1_(paired)_contig_5497 | 1956 | phosphoglycerate kinase | 124.87 | 23.46668 | UP | 3.99E-19 |
| Nonyellowing2_no_rRNA.1_(paired)_contig_52806 | 878 | fructose-1,6-bisphosphatase | 46.09333 | 3.001389 | UP | 3.65E-11 |
| Nonyellowing2_no_rRNA.1_(paired)_contig_37858 | 1695 | sugar transport protein 13 | 60.2 | 9.118907 | UP | 7.60E-11 |
| Nonyellowing2_no_rRNA.1_(paired)_contig_3674 | 554 | NADH dehydrogenase [ubiquinone] iron-sulfur protein 8 | 7.396667 | 31.1951 | DOWN | 3.56E-04 |
| Nonyellowing2_no_rRNA.1_(paired)_contig_35932 | 812 | phototropin 2 | 22.73333 | 2.70533 | UP | 3.41E-05 |
| Nonyellowing2_no_rRNA.1_(paired)_contig_35037 | 598 | beta-amylase | 74.92 | 10.7879 | UP | 1.02E-13 |
| Nonyellowing2_no_rRNA.1_(paired)_contig_3122 | 3889 | phosphomethylpyrimidine synthase | 58.87667 | 3.869369 | UP | 1.50E-14 |
| Nonyellowing2_no_rRNA.1_(paired)_contig_29370 | 647 | alpha-crystallin domain 32.1 | 22.92 | 0.485568 | UP | 9.01E-07 |
| Nonyellowing2_no_rRNA.1_(paired)_contig_28063 | 841 | cupin domain-containing protein | 17.79333 | 2.703213 | UP | 6.56E-04 |
| Nonyellowing2_no_rRNA.1_(paired)_contig_24893 | 1407 | protein high chlorophyll fluorescence 101 [ | 15.28333 | 1.886304 | UP | 5.47E-04 |
| Nonyellowing2_no_rRNA.1_(paired)_contig_24492 | 1247 | uncharacterized protein | 26.32667 | 0.91062 | UP | 5.88E-08 |
| Nonyellowing2_no_rRNA.1_(paired)_contig_19007 | 893 | bidirectional sugar transporter SWEET11 | 40.27 | 0.765693 | UP | 3.79E-12 |
| First_Contig832 | 248 | glucose-1-phosphate adenylyltransferase large subunit 3 | 0 | 25.04385 | DOWN | 5.24E-07 |
| First_Contig671 | 268 | phosphoglucomutase 3 | 0 | 19.81926 | DOWN | 2.40E-05 |
| First_Contig2392 | 1491 | ubiquitin-conjugating enzyme E2 13 | 0.093333 | 52.95745 | DOWN | 1.39E-14 |
| Starch and sucrose metabolism | | | | | | |
| Nonyellowing2_no_rRNA.1_(paired)_contig_7907 | 1274 | fructose-1,6-bisphosphatase | 60.11 | 3.616089 | UP | 4.03E-15 |
| Nonyellowing2_no_rRNA.1_(paired)_contig_58260 | 687 | sugar transport protein 13 | 40.24333 | 6.844802 | UP | 1.34E-07 |
| Nonyellowing2_no_rRNA.1_(paired)_contig_57870 | 875 | beta-amylase | 20.04 | 2.282414 | UP | 1.13E-04 |
| Nonyellowing2_no_rRNA.1_(paired)_contig_5508 | 1203 | fructose-bisphosphate aldolase 1 | 124.3233 | 1.256722 | UP | 1.53E-36 |
| Nonyellowing2_no_rRNA.1_(paired)_contig_5497 | 1956 | phosphoglycerate kinase | 124.87 | 23.46668 | UP | 3.99E-19 |
| Nonyellowing2_no_rRNA.1_(paired)_contig_5497 | 1956 | phosphoglycerate kinase | 124.87 | 23.46668 | UP | 3.99E-19 |
| Nonyellowing2_no_rRNA.1_(paired)_contig_52806 | 878 | fructose-1,6-bisphosphatase | 46.09333 | 3.001389 | UP | 3.65E-11 |
| Nonyellowing2_no_rRNA.1_(paired)_contig_37858 | 1695 | sugar transport protein 13 | 60.2 | 9.118907 | UP | 7.60E-11 |
| Nonyellowing2_no_rRNA.1_(paired)_contig_3674 | 554 | NADH dehydrogenase [ubiquinone] iron-sulfur protein 8 | 7.396667 | 31.1951 | DOWN | 3.56E-04 |
| Nonyellowing2_no_rRNA.1_(paired)_contig_35932 | 812 | phototropin 2 | 22.73333 | 2.70533 | UP | 3.41E-05 |
| Nonyellowing2_no_rRNA.1_(paired)_contig_35037 | 598 | beta-amylase | 74.92 | 10.7879 | UP | 1.02E-13 |
| Nonyellowing2_no_rRNA.1_(paired)_contig_3122 | 3889 | phosphomethylpyrimidine synthase | 58.87667 | 3.869369 | UP | 1.50E-14 |
| Nonyellowing2_no_rRNA.1_(paired)_contig_29370 | 647 | alpha-crystallin domain 32.1 | 22.92 | 0.485568 | UP | 9.01E-07 |
| Nonyellowing2_no_rRNA.1_(paired)_contig_28063 | 841 | cupin domain-containing protein | 17.79333 | 2.703213 | UP | 6.56E-04 |
| Nonyellowing2_no_rRNA.1_(paired)_contig_24893 | 1407 | protein high chlorophyll fluorescence 101 | 15.28333 | 1.886304 | UP | 5.47E-04 |
| Nonyellowing2_no_rRNA.1_(paired)_contig_24492 | 1247 | uncharacterized protein | 26.32667 | 0.91062 | UP | 5.88E-08 |
| Nonyellowing2_no_rRNA.1_(paired)_contig_19007 | 893 | bidirectional sugar transporter SWEET11 | 40.27 | 0.765693 | UP | 3.79E-12 |
| First_Contig832 | 248 | glucose-1-phosphate adenylyltransferase large subunit 3 | 0 | 25.04385 | DOWN | 5.24E-07 |
| First_Contig671 | 268 | phosphoglucomutase 3 | 0 | 19.81926 | DOWN | 2.40E-05 |
| First_Contig2392 | 1491 | ubiquitin-conjugating enzyme E2 13 | 0.093333 | 52.95745 | DOWN | 1.39E-14 |
| Other transcripts | | | | | | |
| First_Contig2662 | 448 | translation initiation factor eIF-5A | 194.8426245 | 0 | up | 5.11E-55 |
| First_Contig230 | 228 | Root hair defective 3 GTP-binding protein (RHD3) | 19.845 | 0 | up | 2.40E-05 |
| Nonyellowing2_no_rRNA.1_(paired)_contig_75978 | 1853 | trans-cinnamate 4-monooxygenase | 23.5966631 | 3.963333 | UP | 2.09E-04 |
| Nonyellowing2_no_rRNA.1_(paired)_contig_5856 | 3228 | putative cinnamoyl-CoA reductase | 13.77 | 0.876054032 | UP | 3.8E-04 |
| Nonyellowing2_no_rRNA.1_(paired)_contig_11713 | 1207 | cinnamyl alcohol dehydrogenase 7 | 94.25667 | 11.50069469 | UP | 1.21E-06 |
| Nonyellowing2_no_rRNA.1_(paired)_contig_34885 | 980 | translationally-controlled tumor protein-like protein | 97.07866298 | 15.78 | UP | 9.83E-15 |
| First_Contig515 | 558 | CLE-like (CLEL) peptides | 43.83211502 | 0.09666 | UP | 4.78E-12 |
