# Supplementary material for: Characterization of the Transcriptome and Gene Expression of Tetraploid Black Locust Cuttings in Response to Etiolation
Source: Genes (Basel). 2017 Nov 24;8(12):345. doi: 10.3390/genes8120345 (PMC5748663; doi:10.3390/genes8120345)
Supplement: Supplementary file 1 [file genes-08-00345-s001.zip › genes-231221_supplementary_final/genes-231221_final_supplementary.pdf]

Supplementary Materials: Characterization of the Transcriptome and Gene Expression of Tetraploid Black Locust Cuttings in Response to Etiolation

Nan Lu [1,2,†], Li Dai [1,†], Zijing Luo [1], Shaoming Wang [3], Yanzhong Wen [4], Hongjing Duan [1], Rongxuan Hou [1], Yuhan Sun [1], and Yun Li [1,*]

[1] Beijing Advanced Innovation Center for Tree Breeding by Molecular Design, National Engineering Laboratory for Tree Breeding, Key Laboratory of Genetics and Breeding in Forest Trees and Ornamental Plants of Ministry of Education, College of Biological Sciences and Technology, Beijing Forestry University, Beijing 100083, China; ln_890110@163.com (N.L.); daili.jiayou@163.com (L.D.); luozijingbjfu@163.com (Z.L.); duan673356712@126.com (H.D.); hrxvshzy@163.com (R.H.); syh831008@163.com (Y.S.)

[2] State Key Laboratory of Forest Genetics and Tree Breeding, Key Laboratory of Tree Breeding and Cultivation of State Forestry Administration, Research Institute of Forestry, Chinese Academy of Forestry, Beijing 100091, China

[3] The State-Owned Luoning County Lv Cun Forest Farm, Luoning 471711, China; lclc873@163.com

[4] The State-Owned Ji County Nursery of Forests, Linfen 042200, China; jxzzywyz@163.com.

* Correspondence: yunli@bjfu.edu.cn; Tel.: +86-10-6233-6094

† These authors contributed equally to this work.

Table S1. Summary of sequencing and assembly.

Sample ID	Replicates	Raw read	Quality trimmed	Adaptor trimmed	rRNA trimmed	Clean ratio
Non-etiolated treatment (control)	1	62,315,544	61,876,804	60,479,091	59,050,378	94.76%
	2	87,977,154	87,501,884	85,534,080	83,700,310	95.14%
	3	52,642,822	52,318,969	50,943,879	49,830,450	94.66%
Average		67,645,173	67,232,552	65,652,350	64,193,713	94.85%
Etiolated treatment	1	62,385,126	62,060,405	60,612,441	59,321,960	95.09%
	2	61,606,896	55,662,423	31,551,135	29,184,730	47.37%
	3	61,420,516	60,795,194	58,393,997	56,877,804	92.60%
Average		61,804,179	59,506,007	50,185,858	48,461,498	78.35%

Table S2. Summary of the tetraploid *R. pseudoacacia* transcriptome.

Statistic	Count	Total length (bp)	N25 (bp)	N50 (bp)	N75 (bp)	Average length	Longest (bp)	N%	GC%	Annotation count	Annotation rate
Contigs	113,233	78,059,940	2,015	1,030	474	689	15,626	0.4	37.6	-	-
Primary UniGene	126,350	77,825,120	1,735	880	424	616	15,626	0.4	37.6	-	-
Final UniGene	107,564	81,197,064	2,221	1,158	536	755	16,999	3.84	36.28	52,590	48.89%

Table S3. Categorization of tetraploid *R. pseudoacacia* with KEGG biochemical pathways.

Pathway ID	KEGG categories	Mapped_KO	Unigene no.	Ratio of no. (%)
ko01100	Metabolic pathways	454	681	2.35
ko01110	Biosynthesis of secondary metabolites	230	376	1.30
ko01130	Biosynthesis of antibiotics	109	169	0.58
ko01120	Microbial metabolism in diverse environments	88	155	0.53
ko04141	Protein processing in endoplasmic reticulum	40	114	0.39
ko01200	Carbon metabolism	63	106	0.37
ko00500	Starch and sucrose metabolism	31	105	0.36
ko01230	Biosynthesis of amino acids	62	102	0.35
ko05169	Epstein-Barr virus infection	30	87	0.30
ko03010	Ribosome	57	80	0.28
ko00620	Pyruvate metabolism	24	77	0.27
ko00190	Oxidative phosphorylation	43	68	0.23
ko00230	Purine metabolism	45	67	0.23
ko00010	Glycolysis / Gluconeogenesis	28	63	0.22
ko04144	Endocytosis	27	55	0.19
ko03013	RNA transport	42	54	0.19
ko00195	Photosynthesis	15	52	0.18
ko04075	Plant hormone signal transduction	24	51	0.18
ko00940	Phenylpropanoid biosynthesis	15	47	0.16
ko00240	Pyrimidine metabolism	34	46	0.16
ko03018	RNA degradation	30	43	0.15
ko03015	mRNA surveillance pathway	30	42	0.14
ko05230	Central carbon metabolism in cancer	11	42	0.14
ko04145	Phagosome	18	41	0.14
ko04151	PI3K-Akt signalling pathway	22	40	0.14
ko03040	Spliceosome	36	39	0.13
ko05203	Viral carcinogenesis	24	39	0.13
	Others		3408	11.75

Ratio of no.: number of unigenes/total genes in all mapped KO pathways.

Table S7. Transcript expression level of part differentially expressed genes.

Unigene id	Unigene_length	Ath BLAST	E RPKM	NE RPKM	UP/DOWN regulated	*p*-value
		Hormone metabolism and response				
Nonyellowing2_no_rRNA.1_(paired)_contig_3822	2796	Thermospermine synthase ACAULIS5	25.01667	3.188976	UP	2.38E-05
Nonyellowing2_no_rRNA.1_(paired)_contig_57734	268	lecithin retinol acyltransferase domain protein	0.543333	14.55347	DOWN	5.58E-04
First_Contig51	4191	auxin response factor 6	0.026667	14.77261	DOWN	5.58E-04
Nonyellowing2_no_rRNA.1_(paired)_contig_13600	2347	cytokinin oxidase/dehydrogenase 6	9.7	51.71855	DOWN	1.36E-07
Nonyellowing2_no_rRNA.1_(paired)_contig_41663	1921	cytokinin dehydrogenase 1	1.236667	17.3687	DOWN	4.43E-04
Nonyellowing2_no_rRNA.1_(paired)_contig_78841	2164	auxin efflux carrier-like protein	21.46333	0.129654	UP	1.78E-06
Nonyellowing2_no_rRNA.1_(paired)_contig_3822	2796	Thermospermine synthase ACAULIS5	25.01667	3.188976	UP	2.38E-05
Nonyellowing2_no_rRNA.1_(paired)_contig_8602	1772	IAA-amino acid hydrolase IAR3	51.61333	5.692749	UP	4.74E-11
Nonyellowing2_no_rRNA.1_(paired)_contig_22193	246	1-aminocyclopropane-1-carboxylate oxidase	111.7833	21.01783	UP	4.38E-17
Nonyellowing2_no_rRNA.1_(paired)_contig_18123	381	1-aminocyclopropane-1-carboxylate oxidase	70.42333	15.20754	UP	2.37E-10
Nonyellowing2_no_rRNA.1_(paired)_contig_8411	966	gibberellin-regulated protein 14	30.49	1.668845	UP	3.75E-08
		Photosynthesis				
Nonyellowing2_no_rRNA.1_(paired)_contig_29372	457	light harvesting complex photosystem II subunit 6	37.14333	0.163333	UP	3.02E-11
Nonyellowing2_no_rRNA.1_(paired)_contig_21389	1687	glyceraldehyde 3-phosphate dehydrogenase GAPA2	31.43667	0.276659	UP	1.90E-09
Nonyellowing2_no_rRNA.1_(paired)_contig_22922	961	light-harvesting chlorophyll B-binding protein 3	17.60333	0.156667	UP	2.64E-05
Nonyellowing2_no_rRNA.1_(paired)_contig_22921	1023	light-harvesting chlorophyll B-binding protein 3	60.89667	0.583622	UP	3.63E-18
Nonyellowing2_no_rRNA.1_(paired)_contig_5508	1203	fructose-bisphosphate aldolase 1	124.3233	1.256722	UP	1.53E-36
Nonyellowing2_no_rRNA.1_(paired)_contig_4294	1451	ribulose bisphosphate carboxylase/oxygenase activase	42.25	0.45207	UP	9.49E-13
Nonyellowing2_no_rRNA.1_(paired)_contig_667	1150	chlorophyll a-b binding protein 6	286.44	3.731085	UP	1.99E-83
Nonyellowing2_no_rRNA.1_(paired)_contig_23633	2266	thylakoid rhodanese-like protein	49.26333	0.680224	UP	7.41E-15
Nonyellowing2_no_rRNA.1_(paired)_contig_828	982	chlorophyll a-b binding protein CP26	263.95	4.377162	UP	6.77E-75

Nonyellowing2_no_rRNA.1_(paired)_contig_4273	906	ribulose bisphosphate carboxylase/oxygenase activase	111.56	1.911763	UP	1.40E-32
Nonyellowing2_no_rRNA.1_(paired)_contig_34263	1487	chloroplast stem-loop binding protein	14.04	0.322847	UP	3.02E-11
Nonyellowing2_no_rRNA.1_(paired)_contig_6381	457	light harvesting complex photosystem II subunit 6	112.67	4.431623	UP	1.90E-09
Nonyellowing2_no_rRNA.1_(paired)_contig_6226	879	PSI type III chlorophyll a/b-binding protein	300.1233	12.82617	UP	2.64E-05
Nonyellowing2_no_rRNA.1_(paired)_contig_5239	834	photosystem I reaction center subunit D-2	137.3167	6.270678	UP	3.63E-18
Nonyellowing2_no_rRNA.1_(paired)_contig_5511	1756	phosphoribulokinase	73.8	3.535588	UP	1.53E-36
Nonyellowing2_no_rRNA.1_(paired)_contig_8339	4829	magnesium chelatase subunit H	40.86667	2.952852	UP	9.49E-13
Nonyellowing2_no_rRNA.1_(paired)_contig_2104	911	ferredoxin-NADP(+)-oxidoreductase 1	65.95	5.616228	UP	1.99E-83
Nonyellowing2_no_rRNA.1_(paired)_contig_9400	784	photosystem II subunit P-1	202.33	17.82207	UP	7.41E-15
Nonyellowing2_no_rRNA.1_(paired)_contig_4324	1411	photosystem II subunit Q-2	77.91	7.078999	UP	6.77E-75
Nonyellowing2_no_rRNA.1_(paired)_contig_5684	1096	photosystem I subunit F	158.7967	14.55142	UP	1.40E-32
Nonyellowing2_no_rRNA.1_(paired)_contig_1769	1140	ferretin 1	461.2533	43.42731	UP	1.96E-04
Nonyellowing2_no_rRNA.1_(paired)_contig_13245	942	plastid-lipid associated protein PAP / fibrillin family protein	35.31	4.049767	UP	4.29E-29
Nonyellowing2_no_rRNA.1_(paired)_contig_24893	1407	protein high chlorophyll fluorescence 101	15.28333	1.886304	UP	2.53E-76
Nonyellowing2_no_rRNA.1_(paired)_contig_8193	939	chlorophyllase 2	23.87	3.533976	UP	1.38E-34
Nonyellowing2_no_rRNA.1_(paired)_contig_15397	439	photosystem II reaction center W protein	36.88	5.648376	UP	7.27E-19
Nonyellowing2_no_rRNA.1_(paired)_contig_14744	272	photosystem II subunit P-1	49.57	7.86122	UP	4.41E-10
Nonyellowing2_no_rRNA.1_(paired)_contig_1758	1182	oxygen-evolving enhancer protein 1-2	388.25	62.61781	UP	7.67E-15
Nonyellowing2_no_rRNA.1_(paired)_contig_3923	811	photosystem I reaction center subunit N	230.05	39.72191	UP	2.01E-43
		Transcription factor				
Nonyellowing2_no_rRNA.1_(paired)_contig_1254	1626	CCCH-type zinc finger protein SOMNUS	32.56	0.642097	UP	9.53E-10
Nonyellowing2_no_rRNA.1_(paired)_contig_35288	818	NAC domain containing protein 80	15.11	1.200759	UP	5.47E-04
Nonyellowing2_no_rRNA.1_(paired)_contig_8331	1158	zinc finger protein STZ/ZAT10	151.8733	19.73202	UP	3.98E-28
Nonyellowing2_no_rRNA.1_(paired)_contig_9996	1400	dehydration-responsive element-binding protein 1F	21.19333	3.187769	UP	2.34E-04
Nonyellowing2_no_rRNA.1_(paired)_contig_10146	1389	Putative GATA transcription factor 22	53.59667	9.966322	UP	3.80E-09
		Signaling passway				
Nonyellowing2_no_rRNA.1_(paired)_contig_12304	1365	protein NDR1/HIN1-like 10	102.3667	13.45648	UP	3.19E-19
Nonyellowing2_no_rRNA.1_(paired)_contig_30473	1200	aluminum induced protein with YGL and LRDR motifs	278.06	37.28318	UP	1.25E-49
Nonyellowing2_no_rRNA.1_(paired)_contig_1729	2061	cytochrome b6-f complex iron-sulfur subunit	30.45	4.34088	UP	5.14E-06

Nonyellowing2_no_rRNA.1_(paired)_contig_31067	200	ABC transporter G family member 40	4.7	25.99194	DOWN	2.27E-04
Nonyellowing2_no_rRNA.1_(paired)_contig_34557	918	sigma factor binding protein 1	31.80333	7.558408	UP	5.36E-05
Nonyellowing2_no_rRNA.1_(paired)_contig_22729	1420	LRR receptor-like serine/threonine-protein kinase GSO1	7.016667	39.09963	DOWN	7.22E-06
Nonyellowing2_no_rRNA.1_(paired)_contig_3532	1008	uncharacterized protein	7.77	44.01009	DOWN	5.54E-07
Nonyellowing2_no_rRNA.1_(paired)_contig_34885	980	translationally-controlled tumor protein-like protein	15.78	97.07866	DOWN	9.83E-15
Nonyellowing2_no_rRNA.1_(paired)_contig_57734	268	lecithin retinol acyltransferase domain protein	0.543333	14.55347	DOWN	5.58E-04
First_Contig21	3419	CHY and CTCHY and RING-type zinc finger protein	5.623333	300.472	DOWN	1.88E-77
First_Contig421	839	leucine-rich receptor-like protein kinase	0.256667	15.52535	DOWN	2.99E-04
Nonyellowing2_no_rRNA.1_(paired)_contig_42991	2312	flavin-dependent monooxygenase 1	0.14	47.03213	DOWN	3.58E-13
Nonyellowing2_no_rRNA.1_(paired)_contig_11601	1111	L-type lectin-domain containing receptor kinase IX.1	0.876667	667.9659	DOWN	1.59E-04
First_Contig628	218	copper-transporting ATPase RAN1	0	16.50114	DOWN	-
First_Contig3256	975	uncharacterized protein	0	372.0184	DOWN	-
		Stress and wound respond				
Nonyellowing2_no_rRNA.1_(paired)_contig_21346	971	12-oxophytodienoate reductase 2	43.41	1.561867	UP	6.83E-12
Nonyellowing2_no_rRNA.1_(paired)_contig_6523	1614	proline dehydrogenase 2	49.77	10.86958	UP	1.03E-07
Nonyellowing2_no_rRNA.1_(paired)_contig_6099	1023	12-oxophytodienoate reductase 2	83.81	20.82769	UP	4.94E-11
Nonyellowing2_no_rRNA.1_(paired)_contig_6764	334	probable aldo-keto reductase 4	25.99	107.9171	DOWN	3.63E-12
Nonyellowing2_no_rRNA.1_(paired)_contig_10180	657	chitinase-like protein 2	29.73333	146.4826	DOWN	2.18E-18
Nonyellowing2_no_rRNA.1_(paired)_contig_5106	578	lipoxygenase 1	114.31	607.7471	DOWN	6.23E-76
Nonyellowing2_no_rRNA.1_(paired)_contig_75978	1853	trans-cinnamate 4-monooxygenase	3.963333	23.59666	DOWN	2.09E-04
Nonyellowing2_no_rRNA.1_(paired)_contig_52645	2000	cytochrome P450, family 711, subfamily A, polypeptide 1	9.2	62.09315	DOWN	4.49E-10
Nonyellowing2_no_rRNA.1_(paired)_contig_12914	1787	lipoxygenase 1	14.31333	103.534	DOWN	1.13E-16
First_Contig24	2084	lipoxygenase 1	0.036667	15.64174	DOWN	2.99E-04
Nonyellowing2_no_rRNA.1_(paired)_contig_17233	3172	lipoxygenase 2	15.21	0.663333	UP	1.01E-04
Nonyellowing2_no_rRNA.1_(paired)_contig_27964	1908	glutamine synthetase 2	37.27667	3.521123	UP	1.82E-08
Nonyellowing2_no_rRNA.1_(paired)_contig_75389	292	heat shock protein 90.1	0.483333	23.0682	DOWN	1.88E-06
Nonyellowing2_no_rRNA.1_(paired)_contig_23152	205	heat shock protein 70-4	7.783333	39.37533	DOWN	7.22E-06
First_Contig723	249	heat shock protein 70-4	1.00E-05	14.16462	DOWN	0.000558
Nonyellowing2_no_rRNA.1_(paired)_contig_42123	917	heat shock cognate protein 70-1	15.72	71.15462	DOWN	5.12E-09

Nonyellowing2_no_rRNA.1_(paired)_contig_97425	262	heat shock cognate protein 70-1	1.38	64.69076	DOWN	1.49E-16
Nonyellowing2_no_rRNA.1_(paired)_contig_79487	517	heat shock cognate protein 70-1	0.066667	67.1257	DOWN	1.40E-18
Nonyellowing2_no_rRNA.1_(paired)_contig_88924	390	heat shock cognate protein 70-1	1.00E-05	23.09532	DOWN	1.88E-06
Nonyellowing2_no_rRNA.1_(paired)_contig_10854	1628	peroxisomal (S)-2-hydroxy-acid oxidase GLO1	93.22	0.55	UP	1.61E-28
Nonyellowing2_no_rRNA.1_(paired)_contig_30394	1166	peroxiredoxin Q	14.53	0.334102	UP	0.000196
Nonyellowing2_no_rRNA.1_(paired)_contig_11805	614	peroxidase 54	226.29	44.06339	UP	6.48E-33
Nonyellowing2_no_rRNA.1_(paired)_contig_8151	1119	pathogenesis-related thaumatin-like protein	46.63333	11.50389	UP	1.31E-06
Nonyellowing2_no_rRNA.1_(paired)_contig_90435	1220	pathogenesis-related protein 1	107.3333	9.162172	UP	2.15E-23
Nonyellowing2_no_rRNA.1_(paired)_contig_60621	249	kunitz family trypsin and protease inhibitor protein	3.083333	39.42479	DOWN	2.66E-08
Nonyellowing2_no_rRNA.1_(paired)_contig_21986	2600	glycosyl hydrolase 9C2	32.69333	6.93974	UP	1.90E-05
Nonyellowing2_no_rRNA.1_(paired)_contig_11889	750	glycine/proline-rich protein	15.43	0.06	UP	0.000101
Nonyellowing2_no_rRNA.1_(paired)_contig_103	781	dehydration-responsive protein RD22	54.11333	252.4693	DOWN	3.10E-29
Nonyellowing2_no_rRNA.1_(paired)_contig_33260	803	dehydration-responsive protein RD22	1.126667	78.45325	DOWN	1.62E-20
Nonyellowing2_no_rRNA.1_(paired)_contig_90428	257	dehydration-responsive protein RD22	1.00E-05	35.37543	DOWN	8.43E-10
Nonyellowing2_no_rRNA.1_(paired)_contig_10180	657	chitinase-like protein 2	29.73333	146.4826	DOWN	2.18E-18
Nonyellowing2_no_rRNA.1_(paired)_contig_5188	208	chitinase A	2059.44	81.44482	UP	0
Nonyellowing2_no_rRNA.1_(paired)_contig_22292	210	chitinase A	1436.877	66.10533	UP	0
Nonyellowing2_no_rRNA.1_(paired)_contig_7480	764	chitinase A	421.1733	19.90756	UP	-
Nonyellowing2_no_rRNA.1_(paired)_contig_9996	1400	dehydration-responsive element-binding protein 1F	21.19333	3.187769	UP	2.34E-04
Nonyellowing2_no_rRNA.1_(paired)_contig_27366	1737	aspartyl protease-like protein	252.3033	18.01838	UP	1.25E-56
Nonyellowing2_no_rRNA.1_(paired)_contig_92695	1238	aquaporin PIP2-1	0.676667	53.957	DOWN	7.27E-15
Nonyellowing2_no_rRNA.1_(paired)_contig_23084	887	ACT domain-containing protein	53.10667	13.22127	UP	2.76E-07
Nonyellowing2_no_rRNA.1_(paired)_contig_41600	3043	ABC transporter G family member 11	40.97	4.214464	UP	1.51E-08
Nonyellowing2_no_rRNA.1_(paired)_contig_7907	1274	fructose-1,6-bisphosphatase	60.11	3.616089	UP	4.03E-15
Nonyellowing2_no_rRNA.1_(paired)_contig_58260	687	sugar transport protein 13	40.24333	6.844802	UP	1.34E-07
Nonyellowing2_no_rRNA.1_(paired)_contig_57870	875	beta-amylase	20.04	2.282414	UP	1.13E-04
Nonyellowing2_no_rRNA.1_(paired)_contig_5508	1203	fructose-bisphosphate aldolase 1	124.3233	1.256722	UP	1.53E-36
Nonyellowing2_no_rRNA.1_(paired)_contig_5497	1956	phosphoglycerate kinase	124.87	23.46668	UP	3.99E-19
Nonyellowing2_no_rRNA.1_(paired)_contig_5497	1956	phosphoglycerate kinase	124.87	23.46668	UP	3.99E-19
Nonyellowing2_no_rRNA.1_(paired)_contig_52806	878	fructose-1,6-bisphosphatase	46.09333	3.001389	UP	3.65E-11

Nonyellowing2_no_rRNA.1_(paired)_contig_37858	1695	sugar transport protein 13	60.2	9.118907	UP	7.60E-11
Nonyellowing2_no_rRNA.1_(paired)_contig_3674	554	NADH dehydrogenase [ubiquinone] iron-sulfur protein 8	7.396667	31.1951	DOWN	3.56E-04
Nonyellowing2_no_rRNA.1_(paired)_contig_35932	812	phototropin 2	22.73333	2.70533	UP	3.41E-05
Nonyellowing2_no_rRNA.1_(paired)_contig_35037	598	beta-amylase	74.92	10.7879	UP	1.02E-13
Nonyellowing2_no_rRNA.1_(paired)_contig_3122	3889	phosphomethylpyrimidine synthase	58.87667	3.869369	UP	1.50E-14
Nonyellowing2_no_rRNA.1_(paired)_contig_29370	647	alpha-crystallin domain 32.1	22.92	0.485568	UP	9.01E-07
Nonyellowing2_no_rRNA.1_(paired)_contig_28063	841	cupin domain-containing protein	17.79333	2.703213	UP	6.56E-04
Nonyellowing2_no_rRNA.1_(paired)_contig_24893	1407	protein high chlorophyll fluorescence 101 [	15.28333	1.886304	UP	5.47E-04
Nonyellowing2_no_rRNA.1_(paired)_contig_24492	1247	uncharacterized protein	26.32667	0.91062	UP	5.88E-08
Nonyellowing2_no_rRNA.1_(paired)_contig_19007	893	bidirectional sugar transporter SWEET11	40.27	0.765693	UP	3.79E-12
First_Contig832	248	glucose-1-phosphate adenylyltransferase large subunit 3	0	25.04385	DOWN	5.24E-07
First_Contig671	268	phosphoglucomutase 3	0	19.81926	DOWN	2.40E-05
First_Contig2392	1491	ubiquitin-conjugating enzyme E2 13	0.093333	52.95745	DOWN	1.39E-14
		Starch and sucrose metabolism				
Nonyellowing2_no_rRNA.1_(paired)_contig_7907	1274	fructose-1,6-bisphosphatase	60.11	3.616089	UP	4.03E-15
Nonyellowing2_no_rRNA.1_(paired)_contig_58260	687	sugar transport protein 13	40.24333	6.844802	UP	1.34E-07
Nonyellowing2_no_rRNA.1_(paired)_contig_57870	875	beta-amylase	20.04	2.282414	UP	1.13E-04
Nonyellowing2_no_rRNA.1_(paired)_contig_5508	1203	fructose-bisphosphate aldolase 1	124.3233	1.256722	UP	1.53E-36
Nonyellowing2_no_rRNA.1_(paired)_contig_5497	1956	phosphoglycerate kinase	124.87	23.46668	UP	3.99E-19
Nonyellowing2_no_rRNA.1_(paired)_contig_5497	1956	phosphoglycerate kinase	124.87	23.46668	UP	3.99E-19
Nonyellowing2_no_rRNA.1_(paired)_contig_52806	878	fructose-1,6-bisphosphatase	46.09333	3.001389	UP	3.65E-11
Nonyellowing2_no_rRNA.1_(paired)_contig_37858	1695	sugar transport protein 13	60.2	9.118907	UP	7.60E-11
Nonyellowing2_no_rRNA.1_(paired)_contig_3674	554	NADH dehydrogenase [ubiquinone] iron-sulfur protein 8	7.396667	31.1951	DOWN	3.56E-04
Nonyellowing2_no_rRNA.1_(paired)_contig_35932	812	phototropin 2	22.73333	2.70533	UP	3.41E-05
Nonyellowing2_no_rRNA.1_(paired)_contig_35037	598	beta-amylase	74.92	10.7879	UP	1.02E-13
Nonyellowing2_no_rRNA.1_(paired)_contig_3122	3889	phosphomethylpyrimidine synthase	58.87667	3.869369	UP	1.50E-14
Nonyellowing2_no_rRNA.1_(paired)_contig_29370	647	alpha-crystallin domain 32.1	22.92	0.485568	UP	9.01E-07
Nonyellowing2_no_rRNA.1_(paired)_contig_28063	841	cupin domain-containing protein	17.79333	2.703213	UP	6.56E-04
Nonyellowing2_no_rRNA.1_(paired)_contig_24893	1407	protein high chlorophyll fluorescence 101	15.28333	1.886304	UP	5.47E-04
Nonyellowing2_no_rRNA.1_(paired)_contig_24492	1247	uncharacterized protein	26.32667	0.91062	UP	5.88E-08
Nonyellowing2_no_rRNA.1_(paired)_contig_19007	893	bidirectional sugar transporter SWEET11	40.27	0.765693	UP	3.79E-12

First_Contig832	248	glucose-1-phosphate adenylyltransferase large subunit 3	0	25.04385	DOWN	5.24E-07
First_Contig671	268	phosphoglucomutase 3	0	19.81926	DOWN	2.40E-05
First_Contig2392	1491	ubiquitin-conjugating enzyme E2 13	0.093333	52.95745	DOWN	1.39E-14
		Other transcripts				
First_Contig2662	448	translation initiation factor eIF-5A	194.8426245	0	up	5.11E-55
First_Contig230	228	Root hair defective 3 GTP-binding protein (RHD3)	19.845	0	up	2.40E-05
Nonyellowing2_no_rRNA.1_(paired)_contig_75978	1853	trans-cinnamate 4-monooxygenase	23.5966631	3.963333	UP	2.09E-04
Nonyellowing2_no_rRNA.1_(paired)_contig_5856	3228	putative cinnamoyl-CoA reductase	13.77	0.876054032	UP	3.8E-04
Nonyellowing2_no_rRNA.1_(paired)_contig_11713	1207	cinnamyl alcohol dehydrogenase 7	94.25667	11.50069469	UP	1.21E-06
Nonyellowing2_no_rRNA.1_(paired)_contig_34885	980	translationally-controlled tumor protein-like protein	97.07866298	15.78	UP	9.83E-15
First_Contig515	558	CLE-like (CLEL) peptides	43.83211502	0.09666	UP	4.78E-12
